# Room Temperature Structures of Solvated Organic Molecules Enabled by Liquid Cell Electron Diffraction

**DOI:** 10.1021/acscentsci.6c00603

**Published:** 2026-09-22

**Authors:** Niko W. Vlahakis, Krzysztof Konieczny, Cameron W. Flowers, Miguel A. Garcia-Garibay, Jose A. Rodriguez

**Affiliations:** † Department of Chemistry and Biochemistry, University of California, Los Angeles (UCLA), Los Angeles, California 90095, United States; ‡ UCLA-DOE Institute for Genomics and Proteomics; STROBE, NSF Science and Technology Center, University of California, Los Angeles (UCLA), Los Angeles, California 90095, United States

## Abstract

Electron diffraction (MicroED/3DED) enables atomic structure determination from nano- or microcrystals, including dry crystalline powders or mixtures. However, radiation damage and instrument vacuum can limit the interrogation of molecular crystals in solution at room temperature. We now present a strategy for room-temperature MicroED of organic molecular microcrystals suspended in solution, using simple liquid cells: grid sandwiches comprised of two continuous carbon grids that when pressed retain a thin liquid layer between them. In this setting, we find that some room-temperature, hydrated microcrystals are particularly prone to electron beam radiation damage, requiring fast, low dose data collection schemes, direct electron detectors, and multi-crystal data collection. These approaches minimize damage of crystals in liquid cells and facilitate *ab initio* structures at sub-Ångstrom resolution. This tactic enables study of molecular conformations and crystalline phases that may only be accessible in a room-temperature solvated state.

## Graphical abstract

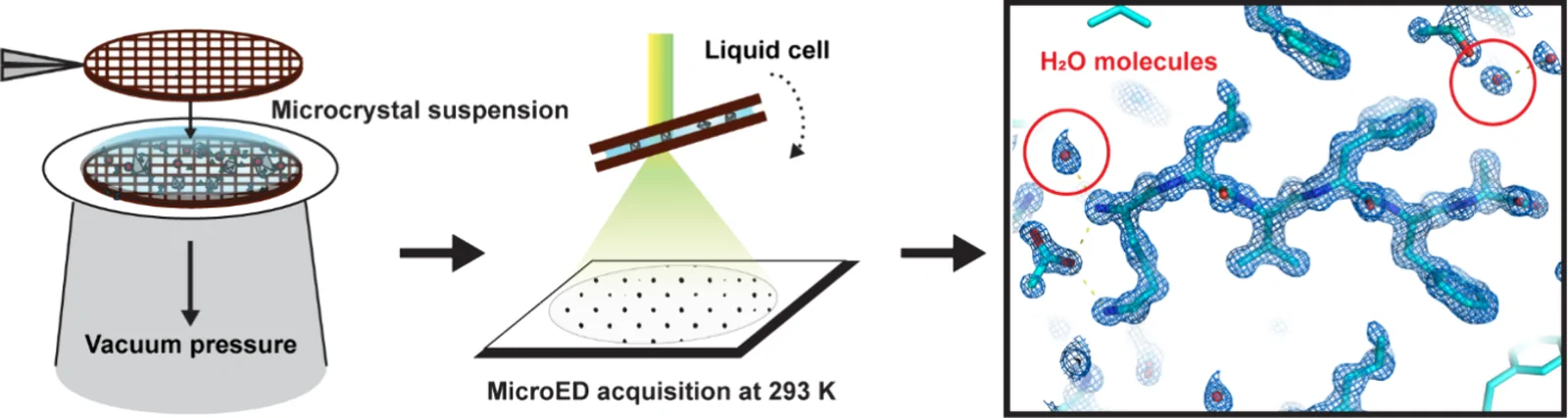

## 1 Introduction

Electron diffraction (MicroED/3DED) is increasingly used to determine atomic structures of small molecules,^1,2^ peptides,^3,4^ and proteins^5,6^ from micro or nano crystals. The electron diffraction experiment typically leverages a transmission electron microscope (TEM) to collect tilt series of diffraction patterns, which are processed using either tools standardly used for single-crystal X-ray diffraction, or programs designed specifically to treat electron diffraction data.^7–9^ However, the method can be challenged by the ionizing energy of the high energy (120–300 keV) electron beam and the less than 10^–5^–10^–8^ Torr of air to which samples are traditionally exposed in the column of a TEM.

For molecular crystals, the structural consequences of energy deposition by a high-energy electron beam are inescapable, since the energy loss of inelastically scattered electrons far exceeds the dissociation energy of a typical molecular bond.^10^ For organic molecules, composed primarily of light atoms, bond dissociation energies are typically less than 100 kcal/mol (∼4 eV), which is less than the typical electron energy loss distribution, centered at tens of eV.^10^ Despite this challenge, inorganic or small molecule microcrystals with tightly packed lattices can yield structures when mounted dry on TEM grids at room temperature.^11^ Meanwhile, solvent-rich, electron beam-sensitive crystals such as those of organic molecule hydrates or biomolecules benefit from frozen-hydrated sample preparation of the kind typically used for cryo-electron microscopy (CryoEM) experiments.^9,12^ Where solvents can be induced to form glassy ice, vitrification helps preserve the integrity of crystals with high solvent content when they are placed under vacuum.^13,14^ This is one of the principal reasons why nearly all MicroED structures of macromolecules have been determined from frozen-hydrated crystals at 100 K.^13,14^ In the unlikely case that a solvated molecular crystal diffracts dry within the TEM, it remains vulnerable to radiolytic damage by the electron beam, which can further impact its crystallinity.^15–20^ Importantly, cryogenic sample preservation presents a challenge when higher temperature insights are sought, for both structural chemistry and structural biology.^21,22^

Recent advances in electron diffraction, in particular, the use of direct electron detectors (DEDs) for rapid, low-dose data collection, have helped overcome or mitigate electron beam damage and led to the determination of ultra-low fluence structures of beam sensitive specimens.^15,23^ In parallel, serial X-ray diffraction data acquisition, where many still diffraction images from unrelated crystal orientations are merged to form a single dataset, has been used in conjunction with room temperature crystallography to limit the impact of radiation damage on the acquired data.^9,24,25^ Analogous serial or serial-like data collection strategies, where either stills or short tilt series wedges are collected and merged together, have been successfully applied to structure determination by electron diffraction,^26–28^ but have not been applied to room temperature, hydrated specimens. Emerging data collection platforms are increasingly streamlining and automating that process, and anecdotal efforts hint at the promise of room temperature structures from crystals in solution.^29–31^

With the goal of expanding the applicability of electron diffraction to room temperature, solvated, dose-sensitive samples, we now determine organic molecular structures *ab initio* from microcrystals held at room temperature in TEM liquid cells. Our simple approach takes advantage of thin carbon layer encapsulation of samples,^32^ whereby we place slurries of microcrystals in a liquid cell (LC) comprised of two continuous carbon grids, analogous to other LC devices used for TEM.^31,33–36^ A thin liquid film remains between the two grids during examination in the TEM, such that diffraction may be acquired from microcrystals in a hydrated state. Low dose data acquisition by DEDs is readily applied to mitigate the high propensity for beam-induced damage under these conditions. In this modality, small wedges of data^26^ tailored to a sample’s characteristic intensity decay rate are automatically collected from dozens of crystals. For some more robust samples, radiolytic damage is insufficient to thwart structure determination, even when using conventional MicroED data collection approaches that rely on scintillator-based detectors. Overall, five atomic room temperature structures of varied solvated small molecule crystals, pharmaceutical hydrates, and biomolecules, collectively illustrate the accessibility and applicability of room temperature liquid cell (LC) MicroED/3DED.

## 2 Results

### 2.1 MicroED of Room Temperature Crystals in Carbon Membrane Liquid Cells

We implemented a strategy for enclosing crystal slurries in LCs for room temperature imaging and diffraction with the aid of pressure-assisted devices^37^ for making LC grid sandwiches.^35^ The approach avoids specialized LC equipment, and can be easily implemented as part of conventional TEM workflows. In our scheme, a glow-discharged, continuous-carbon film TEM grid is placed on filter paper sitting atop a vacuum line, and ∼1 microliter of microcrystal suspension are pipetted onto the grid (Figure 1A). A second TEM grid is placed on top of the first with their squares closely aligned (Figure 1B). Gentle pressure is further exerted downward on the grids by means of a uniform surface such as a parafilm-wrapped glass slide or filter paper to facilitate any excess liquid being wicked out of the sides of the cell and pulled through the bottom filter paper into the vacuum line. This leaves a thin layer of liquid containing the microcrystal slurry trapped between the two grids (Figure 1C). While high vacuum grease can be optionally applied to the circumference of the LC to reinforce its seal (Figure S1A-E), we found capillary action could suffice to induce a seal and keep the liquid layer intact within the TEM (Figure S1F-J).

**Figure 1:**
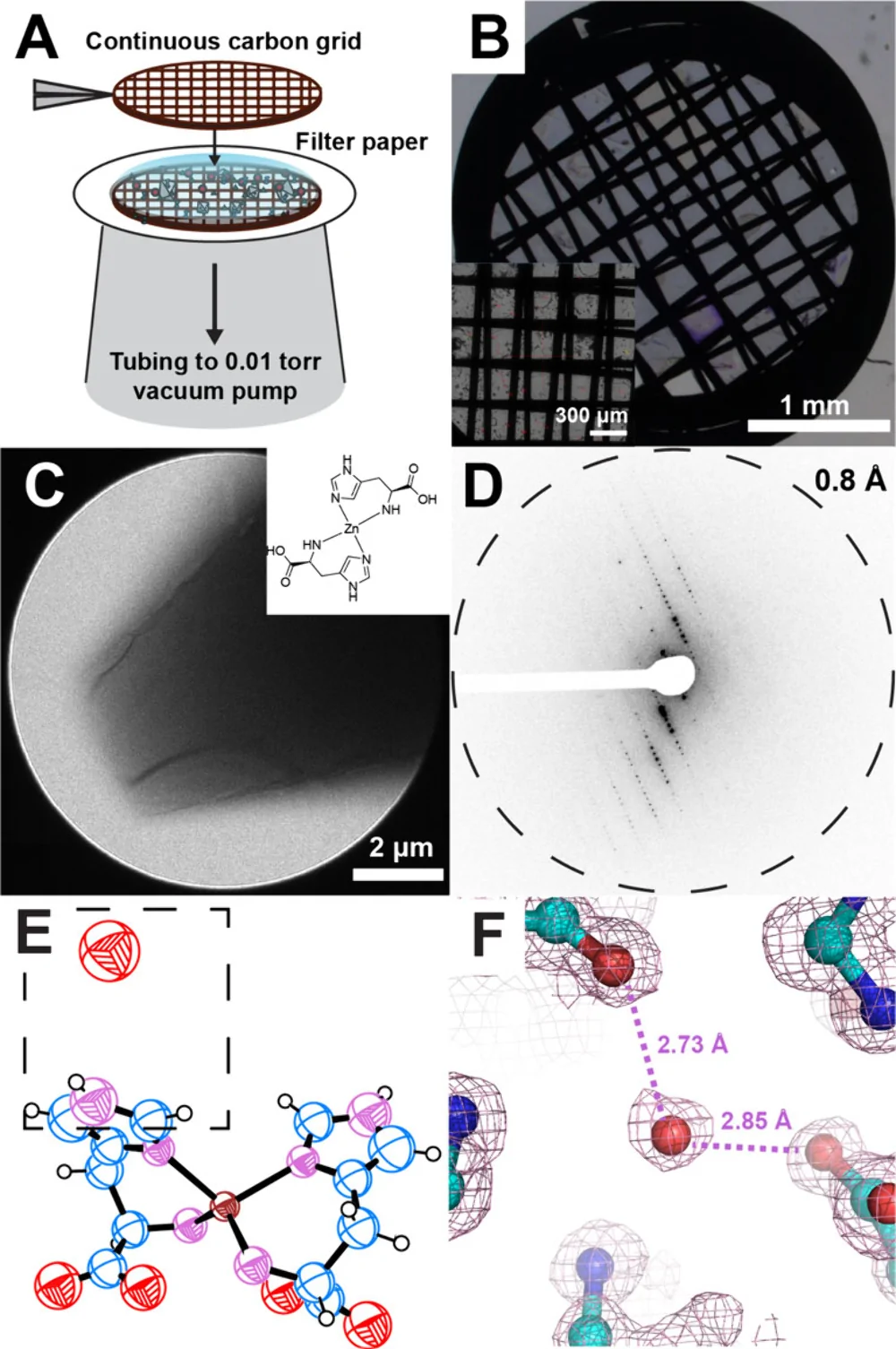
Structure determination by liquid cell, room temperature electron diffraction. Experimental setup for pressure-assisted carbon liquid cell sample preparation (A). A TEM grid is mounted on filter paper atop a vacuum line. Microcrystal slurry is loaded onto the grid over vacuum pressure, and a second grid is aligned and placed on top to form a sandwich held together by capillary action. The sample is inspected by light microscopy (B) and in a TEM atlas (inset), to identify intact liquid regions containing solvated microcrystals (C). Diffraction data is acquired (D), which may yield a structure containing evidence of solvent in the lattice. ORTEP diagram of solvated Zn­(II)-histidine structure at 0.8 Å resolution with water molecule modeled (E), and model superimposed on 1.5σ level electrostatic potential map of the boxed region in E, with hydrogen-bonding contacts with the ordered water molecule annotated (F).

To further investigate the presence of liquid in LC samples prepared by this method, representative LCs were interrogated by electron energy-loss spectroscopy (EELS). LCs loaded with water only contained regions where the liquid front was plainly visible surrounding a dry area by TEM contrast (Figure S2B,D,F,H). Ten random positions in apparently hydrated areas in the proximity of these dry regions were selected, and EELS spectra were recorded from select 2.8 μm diameter illuminated regions using a total fluence of 48 e^‑^/Å^2^ per acquisition (Figure S2E-H). These spectra were compared to equivalent data recorded from illuminated areas within the three dry regions themselves (Figure S2A,C). From each trace the average thickness of the illuminated area in units of inelastic mean-free-path was computed using the log-ratio method (Table S1). An inelastic mean free path of 300 keV electrons in water of 280 nm was assumed based on experimental results from Yesibolati *et al* of thickness measurements in liquid water,^38^ and this value was used to convert the difference in mean thickness measured from wet versus dry sample regions to absolute units. These measurements suggested that in LC samples, a liquid layer of 8 ± 5.8 nm thickness might be expected.

This LC sample preparation strategy suited robust microcrystals of a Zn­(II)-histidine complex, which we routinely prepare from either l-histidine or d-histidine and which crystallize in the enantiomorphic space groups P4_3_2_1_2 or P4_1_2_1_2 respectively. While a large hydrated, cryo-cooled single crystal of Zn­(II)-l-histidine retained two ordered water molecules in the crystal lattice when determined by XRD (Figure S3A-B), no water molecules were detected in a MicroED structure from otherwise equivalent dry, room temperature crystals (Figure S3C–F, Table S2). In contrast, a slurry of Zn­(II)-d-histidine microcrystals suspended in water within a carbon film LC could be loaded into the TEM at room temperature, and a low-magnification atlas collected to locate crystals that occupied intact regions of the carbon sandwich with a discernible liquid layer (Figure 1D). An electron diffraction tilt series from a single crystal of Zn­(II)-d-histidine spanning 90 deg of rotation was acquired, while limiting its exposure to only 0.9 e-/Å^2^ of beam fluence at 200 kV (Figure 1D). That data yielded a structure of the compound with evidence of a single water molecule, in a position consistent with possible hydrogen bonding to neighboring carboxyl groups, in the density map (Figure 1E-F, Table S2). In our experience, the physical and structural properties of these enantiomers are equivalent, save for the space group symmetry, and slight deviations in unit cell dimensions between the XRD and electron diffraction structures of these compounds as measured in this study more likely result from errors in approximation of each experimental geometry rather than any difference in hydration state. The liquid content of representative LC samples of Zn­(II)-l-histidine was also evaluated by EELS using the same strategy as described above. Here, crystals which were apparently hydrated as evidenced by TEM image contrast were identified at low magnification, and positions 3 μm away from each crystal were used to record EELS spectra from 2.8 μm diameter illuminated areas (Figure S4). As the thickness of the crystals themselves (100 nm, at minimum) were too great to record informative EELS data, these crystal-adjacent regions were used as a proxy to estimate the thickness of the liquid layer encompassing the crystals themselves. An equivalent experiment was performed for an LC sample of Zn­(II)-l-histidine that was allowed to fully dry out at ambient conditions for 4 days prior to loading into the TEM (Figure S5). In each case, we measured greater sample thicknesses than from regions in an equivalent, dessicated carbon sandwich sample (Table S3).

### 2.2 Mitigating Electron Beam Damage of Dose-Sensitive Hydrated Crystals

Anticipating most organic molecular crystals to be more beam sensitive than Zn­(II)-histidine crystals, posing a challenge for their interrogation in liquid due to radiolysis of the liquid layer,^39^ we developed a strategy for budgeting electron beam fluence considering both diffraction quality and crystal solvation when acquiring LC MicroED data (Figure S6-8). We established fluence budgets suitable for LC MicroED, initially by irradiating static microcrystals formed by three representative molecules: the hexapeptide d-AVAAGA, Zn­(II)-histidine, and thiostrepton (Figure S6). From a LC grid atlas, we located target crystals in the liquid layer, and acquired dose series of images (Figure S6A,D,G) or diffraction patterns (Figure S6C,F,I). In image series we monitored the impact of electron beam irradiation on the liquid layer surrounding each crystal, and in diffraction series we monitored diffraction quality as a function of fluence. We noted that the maximum fluence threshold for these samples where diffraction beyond 1.2 Å was lost (for d-AVAAGA and Zn­(II)-histidine, referencing Sheldrick’s criterion for successful *ab initio* structure determination by direct methods^40^) roughly mirrored when the net intensity of the 20% brightest reflections had decreased by 10%, and so we considered the latter metric a guide for setting fluence budgets for these three samples (Figure S6). Though some evidence of beam-induced net orientation changes of the crystal lattice was charted in the form of decay profiles of the net reflection intensity from these otherwise static crystals, sometimes inconsistent with a monotonic decline (Figure S6), these were not notably more dramatic than those observed in previous studies performing equivalent measurements on dry and frozen-hydrated crystals.^15^ They are, however, anticipated to occur to some degree in liquid cell samples.^41^ We therefore considered it a viable indicator of crystallinity as a function of fluence. In each case, visible bubbling or drying of the liquid layer surrounding the crystal was also monitored, and the fluence regimes that led to their occurrence were avoided. We therefore estimated d-AVAAGA, Zn­(II)-histidine, and thiostrepton to tolerate roughly 0.45, 0.97, and 0.46 e^‑^/Å^2^ at 200 keV respectively (Figure S6). For the remainder of samples used in this study, all similarly beam-sensitive compared to d-AVAAGA, Zn­(II)-histidine, and thiostrepton, these metrics were approximated and fluence budgets were established from tilt-series data recorded for the purpose of structure determination, as described in the subsequent sections of this report, and all ranged from 0.2 to 1.0 e^‑^/Å^2^ (Table S4). These fluence values were smaller than or on par with what would have been tolerated at cryogenic temperatures (Figure S7). Narrow wedge diffraction tilt series limited by this fluence budget were then automatically recorded from different microcrystals (Figure S8D), and the data from congruent wedges merged to yield atomic structures (Figure S8E-F).

### 2.3 Structure of Triphenyl Acetic Acid s-Block Salt Solvates with Water-Enabled Lattice Contacts

Using LC MicroED samples, we investigated crystals of hydrated molecules in which water molecules facilitate lattice contacts or fill large voids, and thus might be especially prone to destabilization in the TEM vacuum at room temperature. This included triphenyl (3Ph) acetic acid compounds that formed salts with *s*-block metal ions. When drop cast and allowed to dry on a TEM grid, needle-shaped crystals of 3Ph acetic acid potassium salt (Figure 2A) diffracted poorly, showing low resolution, diffuse reflections (Figure S9A-C). By comparison, targeting visibly hydrated microcrystals maintained in a methanol/water (1:9 vol:vol) suspension in a LC (Figure 2B), produced noticeably better single crystal diffraction, surpassing 1.0 Å resolution (Figure 2C, Figure S9D–F). Due to the low triclinic symmetry and preferential orientation adopted by these crystals, merging ∼30 degree wedges of data from 9 separate crystals resulted in a limited, 63.5% completeness dataset. Direct methods nonetheless yielded an *ab initio* structure and ultimately allowed a 1.0 Å resolution structure of the compound to be refined (Figure 2D, Table S5). Although the corresponding acid and base were mixed in 1:1 stoichiometry, the crystal structure revealed 4:1 stoichiometry with 4 acid molecules per potassium cation. This indicated the presence of one carboxylate anion as a formal salt with the potassium cation, complexed with three neutral carboxylic acid molecules within a crystal. The carboxylate anion identity was deduced on the basis of hydrogen bond and COOH/COO^‑^ group geometry. Notably, the electrostatic potential map revealed a peak outside of covalent bonding range of any atoms in 3Ph acetic acid, but 3.03 Å from the potassium ion. This was modeled as a bridging water molecule that facilitated the coordination environment of the metal (Figure 2E).

**Figure 2:**
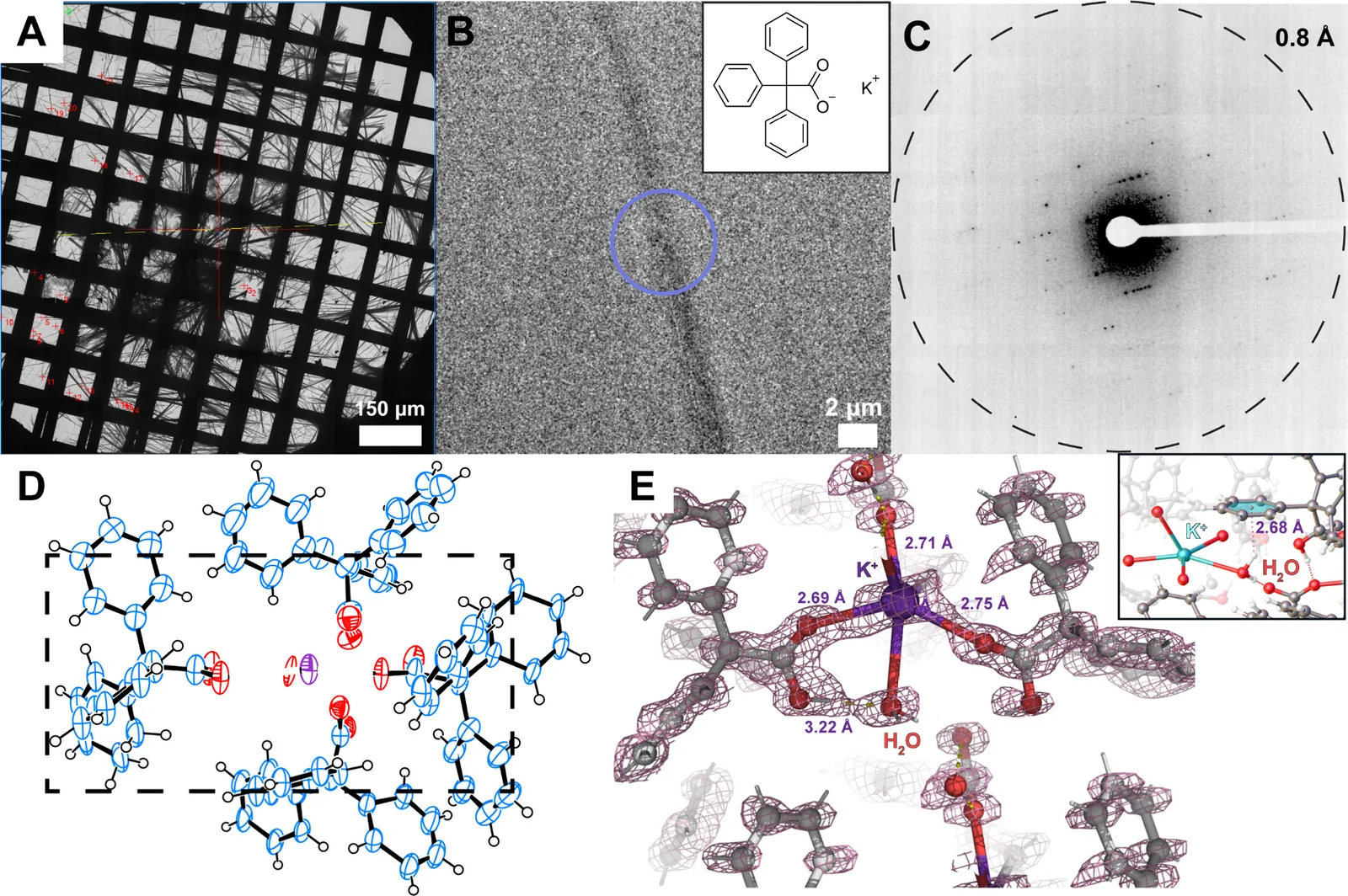
Liquid cell ED of a metal-coordinating compound with water-mediated lattice contacts. TEM atlas of a LC grid sandwich prepared from a slurry of needle-shaped 3Ph acetic acid potassium salt crystals, acquired at room temperature at a TFS Spectra C 300 keV TEM (A). TEM image of a representative hydrated crystal with 2D chemical structure in inset (B) and diffraction pattern (C). ORTEP diagram of the asymmetric unit of a 1.0 Å resolution structure solution of the compound (D), and model superimposed on 1.5σ level electrostatic potential map of the boxed region in D, where strong peaks in the density map indicated a water molecule participating in the coordination environment surrounding the K+ ion (E). Inset of panel E offers a view of the structure highlighting a potential OH-π hydrogen bonding interaction with a participating water molecule.

### 2.4 Applicability of Liquid-Cell MicroED to Pharmaceutical Hydrates

The solvated solid state can be particularly relevant to pharmaceutical compounds, inspiring us to apply LC MicroED to determine room-temperature structures of such compounds. We therefore next assessed the utility of LC MicroED for determining structures of the cephalosporin antibiotic ceftazidime (Figure 3A), which is known to crystallize in a pharmaceutically relevant pentahydrate form, whose five water molecules form hydrogen bonding contacts to stabilize its crystal structure. We hypothesized that these waters might be evacuated under vacuum at room temperature,^42,43^ affecting crystal lattice integrity. Indeed, crystals of ceftazidime pentahydrate drop-cast and dried on a TEM grid diffracted poorly and, when they produced indexable tilt series, maintained a unit cell inconsistent with a pentahydrate polymorph (Figure S10A-B), but matching that of an less hydrated polymorph determined by MicroED at room temperature from dry commercially available ceftazidime monohydrate powder not subject to recrystallization (P2_1_2_1_2_1_; a = 9.46 Å, b = 10.07 Å, c = 25.44 Å) (Figure S10C–F, Table S6). No ordered water molecule was observed in the ceftazidime structure determined from dry crystals, leaving it ambiguous whether the compound remained in the monohydrate form bound to disordered water, or if the water had been evacuated from the lattice by the TEM vacuum.

**Figure 3:**
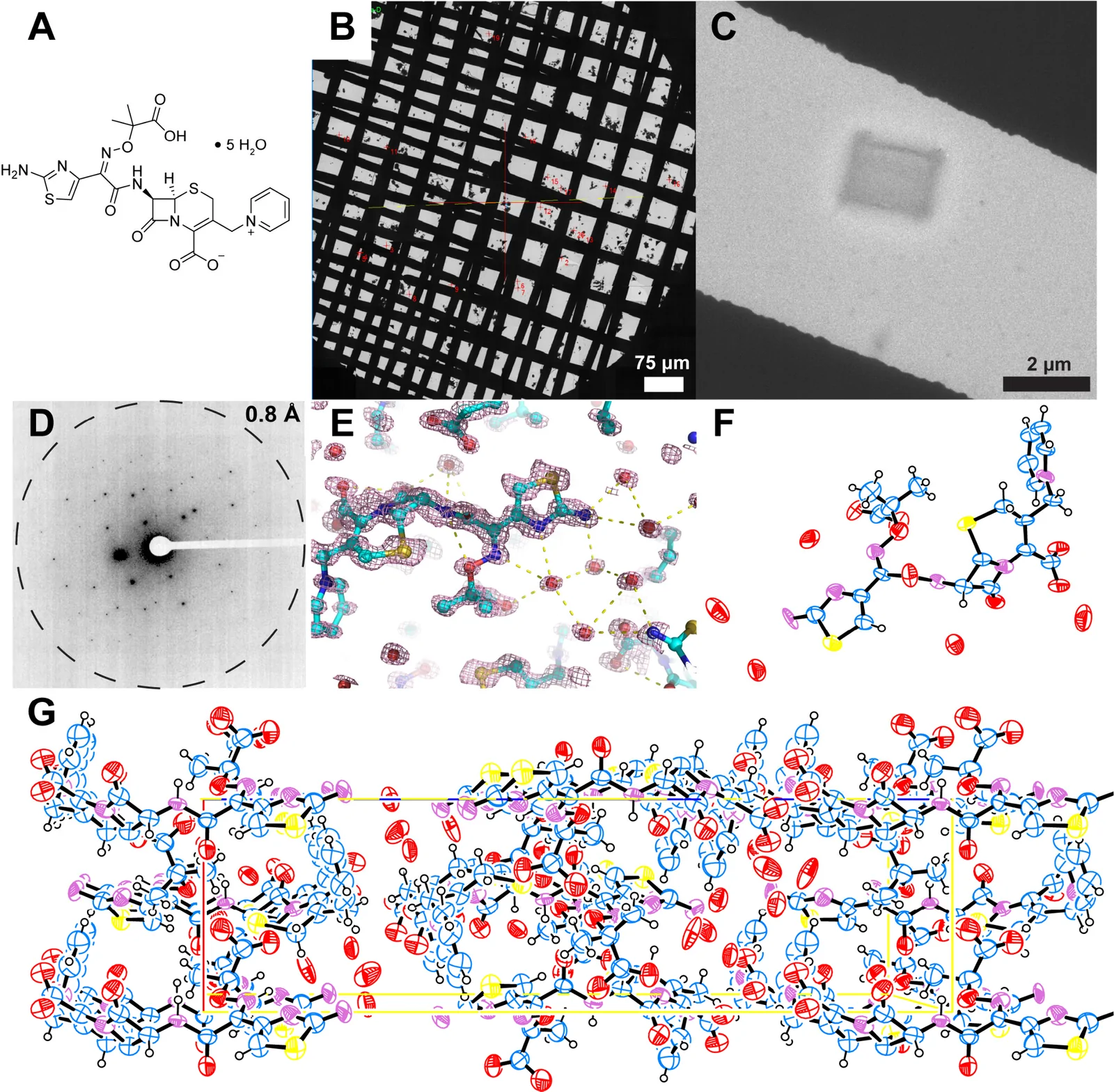
Room temperature liquid cell ED of hydrated pharmaceutical compounds. 2D illustration of ceftazidime, a cephalosporin antibiotic used to treat bacterial infection, in the pharmaceutically relevant pentahydrate form (A). This form was not readily captured by MicroED from dry crystals at room temperature, so LCs containing slurries of ceftazidime pentahydrate microcrystals were prepared. TEM atlas of one such LC grid (B), TEM image of a representative hydrated microcrystal (C), and diffraction pattern from that microcrystal acquired at room temperature (D). 0.8 Å resolution structure solution of the compound, where strong peaks in the density map indicated five water molecules per asymmetric unit in a feasible hydrogen bonding network (E). ORTEP diagram of the asymmetric unit of the structure (F) and ORTEP diagram of a projection of the unit cell along the 010 direction revealing packing of ceftazidime and water within the crystal (G).

By comparison, pentahydrate ceftazidime microcrystals (Figure 3B–C) suspended in a water LC readily diffracted at room temperature (Figure 3D). An 82% complete dataset, achieved by merging wedges of data corresponding to 30-40 degrees of rotation each from eight crystals, was sufficient to determine a 0.8 Å resolution structure of the pentahydrate ceftazidime polymorph by direct methods (Figure 3E-G), with clear electrostatic potential map density indicating the presence of five water molecules in feasible hydrogen-bonding positions (Figure 3F). The unit cell and space group symmetry of the determined crystal form (P2_1_2_1_2_1_; a = 8.92 Å, b = 10.29 Å, c = 31.21 Å) was consistent with the reported XRD structure of the pentahydrate polymorph^42^ (Table S6). An alignment of the refined XRD and MicroED structures showed an overall RMSD of 0.102 for the molecule and 0.024 for the accompanying waters (Figure S11).

### 2.5 Liquid Cell MicroED of Hydrated Polypeptides

We lastly aimed to test the applicability of LC MicroED for the determination of biomolecular structures at room temperature, selecting polypeptides as simple model systems for initial experiments. We applied fluence budgeting to microcrystals of the hexapeptide d-AVAAGA, which have been shown to be beam sensitive but diffract when dry in a TEM.^44^ An atlas of the carbon film LC was first recorded using SerialEM to map microcrystal positions and enable semi-automated diffraction tilt series collection using custom scripts as previously described^23^ (Figure S8A). To assess the hydration of the LC grids, an atlas was collected just following the preparation of a d-AVAAGA crystal grid, which was subsequently left in vacuum for 16 h at 298 K without any additional manipulation, then imaged again. Analysis of images after prolonged vacuum exposure showed tearing of the carbon film suggesting a collapse in the liquid cell (Figure S12). Fifteen crystals suspended in intact LC grids were diffracted to produce ∼30 degree data wedges (Figure S8D). Ten of those wedges were merged into a 92.1% complete dataset with a high-resolution limit of 0.8 Å that was sufficient for *ab initio* structure determination by direct methods (Figure S8E, Table S7). While the refined d-AVAAGA structure showed no coordinated waters (Figure S8F), it demonstrated the feasibility of applying the approach to larger molecules that tolerate our delivered fluence.

We also determined the structure of the amyloidogenic hexapeptide KLVFFA,^45^ which is reported to host several ordered water molecules in its lattice. Crystals of KLVFFA were grown in a solution of 40% isopropanol, 0.3 M ammonium acetate, and 0.1 M Tris HCl pH 8.5, and deposited as a slurry in a LC (Figure 4A). Hydrated KLVFFA crystals in the LC (Figure 4B) diffracted to high resolution without evidence of lattice collapse or disorder (Figures 4C and S13C-D). We collected wedges of ∼30 degrees on 70 crystals at room temperature; each crystal receiving ∼ 0.3 e-/Å^2^ from a 300 keV beam on a scintillator-coupled detector. By contrast, when prepared dry on TEM grids without the LC approach applied, KLVFFA crystals diffracted poorly with disorder indicated by weak and diffuse reflections (Figure S13A-B). Within the LC, KLVFFA crystals robustly diffracted over the ∼ 0.3 e-/Å^2^ duration, and did not ultimately require use of a DED for successful data collection (Figure S13C–D). The recorded tilt series were indexed to a unit cell matching that of the XRD KLVFFA structure, and the reflections from six tilt series were merged to produce a 78% complete dataset. Due to the limited tilt series size achievable for each crystal before onset of decay, and preferential orientation of the crystals which assumed a needle shape, it was challenging to improve data completeness further. While only yielding partial models by direct methods due likely to intermediate data completeness in a non-centrosymmetric space group, the data could be phased by molecular replacement using the XRD structure as a search model and refined at 1.0 Å resolution with somewhat elevated refinement R factors (R_work_/R_free_ = 28.56/31.01) reflective of the incomplete data. A 1.0 Å resolution structure refined from that model showed two coordinated waters and one acetate ion per asymmetric unit around the polypeptide (Figure 4D-E, Table S8). Frozen hydrated crystals of KLVFFA yielded an equivalent structure (Figure S14G), with similar refinement residuals. However, likely due to batch-to-batch variability in crystal quality, the cryogenically preserved crystals diffracted to overall lower resolution and exhibited slightly elevated refinement R factors. Thus, refinement was performed conservatively and limited to 1.5Å (Table S8). Across both peptide structures determined by LC MicroED at room temperature, individual atomic B-factors did not exceed 15.0 Å^2^ (Figure S14, Table S8).

**Figure 4:**
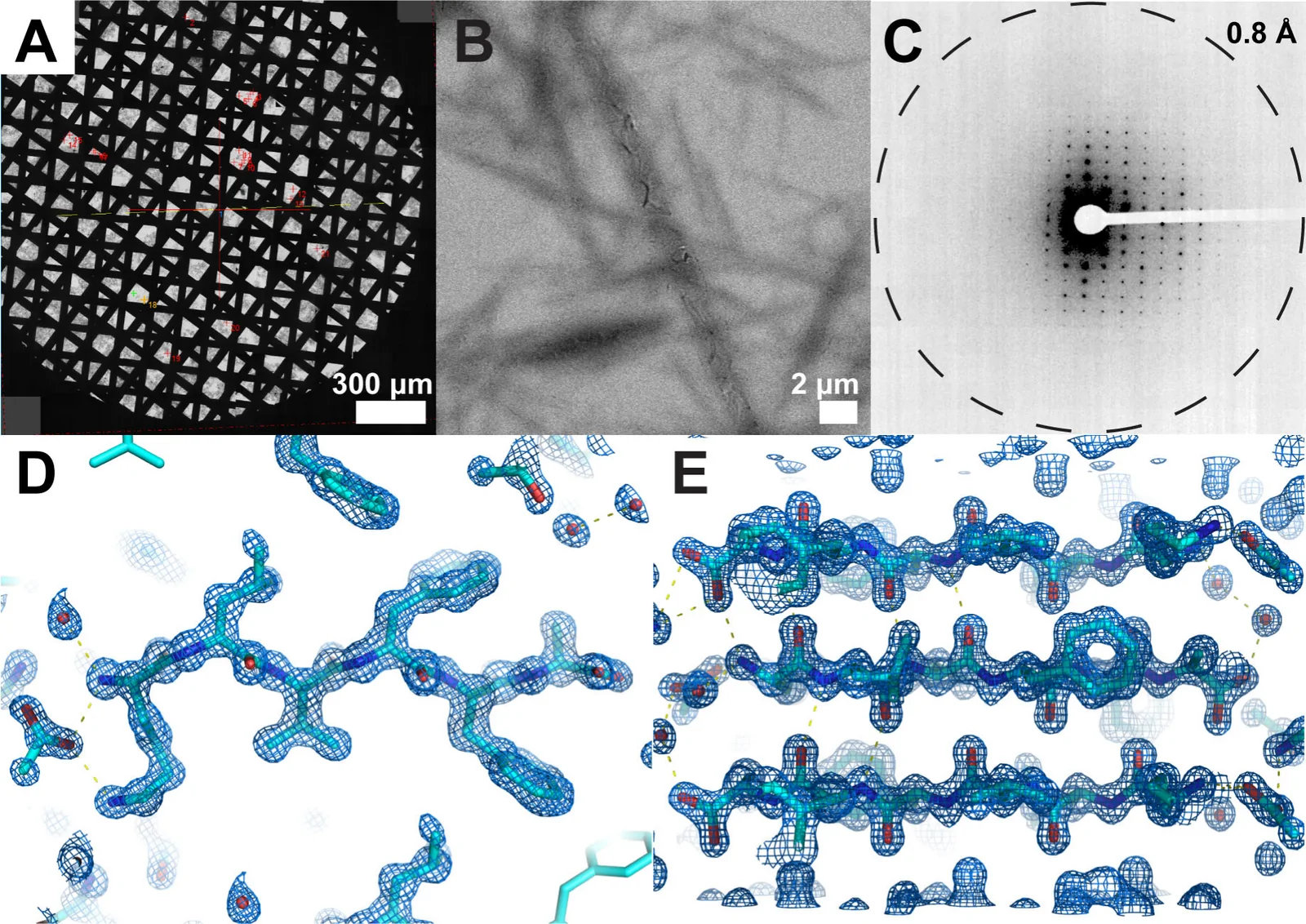
LC MicroED of hydrated KLVFFA peptide crystals. TEM atlas of a carbon LC containing KLVFFA microcrystals in aqueous suspension (A). TEM image of representative hydrated KLVFFA microcrystal (B) and diffraction pattern from the same crystal (C). Structure solution at 1.0 Å resolution of KLVFFA from hydrated, room temperature crystals with ordered water molecules and an acetate ion modeled (D), and alternate view of the structure showing packing between adjacent polypeptide chains (E). Blue mesh indicates the 2F_o_-F_c_ map at 1.5σ level.

## 3 Discussion

Electron diffraction of solvated crystals at room temperature confronts the challenges posed by electron beam-induced damage across all TEM experiments. While high energy electron beams can impact samples in various ways, including electrostatic charging, heating, atomic displacement and ejection, the damage experienced by molecular crystals in solution is an inescapable consequence of secondary electron effects that are exacerbated by the carrier solution.^18,46^ Molecular crystals are further challenged by beam-induced mechanical effects, which can impact the processing of MicroED data.^15^ In spite of these physical limitations, low current densities used in our LC MicroED experiments are sufficient to yield indexable high-resolution diffraction from thin organic molecular crystals.

LC MicroED facilitates structures of pharmaceutical hydrates, suggesting practical applications in structure-informed drug discovery and design. Additionally, its demonstrated applicability to short-chain peptide segments suggests promise for future studies of proteins and protein-small molecule complexes that presently rely on MicroED of cryo-cooled samples.^47^ Measuring electron diffraction from room temperature biomolecular crystals would open a new avenue for structural biology experiments. This opportunity is supported by seminal studies^48,49^ as well as recent efforts demonstrating the possibility of room temperature electron diffraction data collection from hydrated protein crystals.^36^ However, given the high sensitivity of many biological molecules to the electron beam, high throughput serial approaches may be required for their routine structure determination from LCs,^28^ given the success of these approaches on both small- and large-unit cell crystals that do not suffer preferred orientation on a grid.^27,28,44,50^ Additional obstacles might include idiosyncratic chemical environments that emerge within liquid cells, such as the high concentrations of ionic solutes used in graphene-encapsulated solutions in liquid TEM samples,^51^ the impacts of which on MicroED experiments have yet to be explored.

Using a simple strategy for preserving slurries of microcrystals within liquid cells comprised of a carbon film sandwich, we expand the applicability of MicroED by determining room temperature structures of hydrated small molecules, pharmaceutical targets, and simple biomolecules. We find that carbon film LCs are compatible with standard TEMs and accommodate solvents that might otherwise pose a challenge for data collection at liquid nitrogen temperatures, in particular solutions that cannot readily be induced to form glasses. Our results also support the hypothesis that carbon encapsulation of specimens may be radioprotective^46,52^ and that added carbon layer supports can reduce beam-induced motion,^53^ allowing simple but effective dose budgeting to inform the collection and merging of diffraction data across multiple minimally damaged crystals. Even then, the most dose-sensitive samples require that a sufficiently low incident fluence be delivered to each crystallite, and that a concomitantly higher number of smaller wedges of MicroED data be combined to yield a structure.^54^ New methodological developments would be required to offset this dynamic in favor of less damaged structures.

## Supplementary Material



## Data Availability

MicroED structures of Zn­(II)-histidine, 3PhCOOK, ceftazidime, and d-AVAAGA are available under CCDC deposition numbers 2481287 (dry-RT Zn­(II)-histidine), 2521667 (dry-cryo Zn­(II)-histidine), 2481286 (LC-RT Zn­(II)-histidine), 2481288 (3PhCOOK), 2481289 (LC-RT ceftazidime pentahydrate), 2481290 (dry-RT anhydrous ceftazidime), 2481974 (LC-RT d-AVAAGA), and 2521666 (dry-cryo d-AVAAGA). LC-MicroED structure of KLVFFA is available via PDB entry 9Q2V, and cryo MicroED structure of KLVFFA is available via PDB entry 11ZN. Raw diffraction datasets are available under Zenodo entries 1699760, 18200247, 19142231. EELS data is available under Zenodo entry 21302947.

## Notes

Methods, Figures S1–S14, Tables S1–S8, and CheckCIF structure validation reports and responses (PDF)

## §

National Center for Electron Microscopy, Molecular Foundry, Lawrence Berkeley National Laboratory, Berkeley, CA 94720, USA

## Abbreviations

MicroED: microcrystal electron diffraction
3DED: three-dimensional electron diffraction
LC: liquid cell
eV: electron volts
EELS: electron energy-loss spectroscopy.
