# Supplementary material for: Room Temperature Structures of Solvated Organic Molecules Enabled by Liquid Cell Electron Diffraction
Source: ACS Cent Sci. 2026 Sep 22;12(10):1592–600. doi: 10.1021/acscentsci.6c00603 (PMC13720921; doi:10.1021/acscentsci.6c00603)
Supplement: Supplementary file 1 [file oc6c00603_si_001.pdf]

Supplementary Materials for

Room temperature structures of solvated organic molecules enabled by liquid cell electron diffraction

Niko W. Vlahakis[1,2*†], Krzysztof Konieczny[1], Cameron W. Flowers[1,2], Miguel A. Garcia-Garibay[1], Jose A. Rodriguez[1,2*]

[1] Department of Chemistry and Biochemistry; University of California, Los Angeles (UCLA); Los Angeles, CA 90095, USA.

[2] UCLA-DOE Institute for Genomics and Proteomics; STROBE, NSF Science and Technology Center; University of California, Los Angeles (UCLA); Los Angeles, CA 90095, USA.

[†] Present address: National Center for Electron Microscopy, Molecular Foundry, Lawrence Berkeley National Laboratory, Berkeley, CA 94720, USA

Corresponding authors: Niko W. Vlahakis (nwvlahakis@lbl.gov) and Jose A. Rodriguez (jrodriguez@mbi.ucla.edu)

The PDF file includes:

Materials and Methods
Figs. S1 to S14
Tables S1 to S8
CheckCIF structure validation reports and responses
Supplementary references

Other Supplementary Materials for this manuscript include the following:

Datasets deposited on Zenodo
Structures deposited to the PDB and CCDC
PDB structure validation reports

Materials and Methods

Crystallization of samples

Zn(II)-histidine: Zinc hydroxide ($Zn(OH)_2$) was prepared by dissolving Zn(II) chloride in 1 M NaOH at a 1:2 molar ratio. The solution was stirred for 1 hour, during which $Zn(OH)_2$ precipitated as a white solid. This was isolated by vacuum filtration, washed with cold water, and air-dried. 2 molar equivalents L-histidine or D-histidine and 1 molar equivalent $Zn(OH)_2$ were dissolved in separate aqueous solutions, then mixed together and stirred at 50 ^oC for 2 hours. The solution was air-dried until reduced to approximately 25% of its original volume, and left at room temperature. Colorless, tetragonal crystals formed in 1-2 days.

d-AVAAGA: d-AVAAGA was ordered from Genscript and dissolved in water at 10 mg/mL concentration. Crystals were grown by hanging drop vapor diffusion where hanging drops were made from a 1:1 volume ratio of peptide solution to reservoir solution (0.1 M Na citrate pH 6.5, 10% ethanol).

3PhCOOK: The triphenyl acetic acid (97%) and potassium hydroxide were purchased from AmBeed and Fischer Chemicals, respectively. The acid (ca. 20 mg) was dissolved in milliliters of methanol to which 0.3 ml of KOH water solution was added in stoichiometric amount (1:1). The mixture was sonicated and let overnight to dry. The next day formed salt was redissolved in water/methanol mixture (9:1 vol/vol ratio) and let for partial solvent evaporation until needle-shaped colorless crystals were formed in solution.

Ceftazidime pentahydrate: ceftazidime monohydrate was purchased from Sigma and suspended in water at a concentration of 120 mg/mL. The suspension was stirred while 5 M NaOH was added dropwise until reaching pH 7.0, at which point the ceftazidime monohydrate was able to fully dissolve into solution. This solution was transferred to an ice batch and allowed to cool, and 5 M HCl was added dropwise until reaching pH 4.0. The solution was left on ice for 2-3 hours, during which time showers of small rectangular crystals formed.

KLVFFA: 95% pure peptide was ordered from Genscript and dissolved in water at 10 mg/mL concentration. Crystals were first grown by hanging drop vapor diffusion where hanging drops were made from a 1:1 volume ratio of peptide solution to reservoir solution (40% isopropanol, 0.3 M ammonium acetate, 0.1 M Tris HCl pH 8.5). These crystals, which formed as a bundle of needles, were used to seed a crystallization batch of 5 mg/mL KLVFFA dissolved in 40% isopropanol, 0.3 M ammonium acetate, 0.1 M Tris HCl pH 8.5. Within a day, the batch was full of showers of small needle-shaped crystals.

Preparation of TEM liquid cells

Continuous carbon-film copper mesh TEM grids were used for all LC preparations reported in this study. 75, 150, or 200 mesh size grids were used, where it was noted that lower mesh sizes enabled easier alignment between the two grids of the LC and allowed for diffraction from higher tilt angles to be measures, while higher mesh sizes made breaches in the carbon film of the LC less likely. LCs were prepared as follows: tubing connected to a vacuum line was equipped with a modified micropipette tip on its open end, where the end of the pipette tip was cut off to make a wide opening, and a metal mesh was glued to this opening to cover it. The tip of the vacuum line was held upright with a clamp and acted as a platform to rest grids on atop filter paper during sample preparation. 1-1.5 μL of microcrystal slurry was added to the carbon face of a glow-discharged TEM grid resting on this platform, while vacuum was pulled from behind via the vacuum line. A second TEM grid, with the carbon side facing downward toward the sample, was aligned with the first and placed on top of it to create a sandwich. A glad microscope slide was

used to gently apply downward force on the sandwich, allowing excess liquid to be wicked away by the filter paper beneath. LC grids were stored in a chamber under vacuum for 30 minutes to 1 hour prior to insertion into a TEM, to reduce the likelihood of introducing moisture into the TEM column and degrading the vacuum.

Preparation of conventional MicroED samples
Either Zn(II)-L-histidine or Zn(II)-D-histidine microcrystals were dusted onto continuous carbon TEM grids, and inserted without further preparation into the TEM. Room temperature MicroED data was recorded immediately afterwards. For data at cryogenic temperatures, grids prepared in this fashion were loaded into a side-entry cryotransfer holder at room temperature and inserted into the microscope, after which the holder dewar was filled with liquid nitrogen and allowed to equilibrate to roughly 100 K prior to data acquisition.
2 μL of d-AVAAGA microcrystals suspended in their crystallization buffer were dropcast onto glow-discharged continuous carbon TEM grids. The suspension was allowed to incubate on the grid for 1 minute, and then excess solvent was wicked away by contacting the edge of the grid-mounted droplet with a piece of filter paper. These grids were loaded into the TEM in a side-entry cryotransfer holder at room temperature, prior to cooling by filling the holder dewar with liquid nitrogen.
KLVFFA crystals were crystallized identically to crystals prepared for liquid cell experiments.
All samples for hydrated cryoEM experiments were prepared on CF-1.2/1.3-3Cu-50 300 mesh C-flat™ grids. Grids were glow discharged with a PELCO easiGlow system with a plasma current of 15mAmps for 15 sec on both sides. The grids were then mounted on an FEI Vitrobot with 100% humidity at 11°C. KLVFFA crystals were diluted 1:5 in mother liquor directly before applying 1.25 uL of diluted crystal solution to the carbon film side of the grid. The grids were blotted manually using tweezers holding filter paper to contact the opposing face from which the sample was applied for 30 s. Directly following blotting the grid was plunge frozen in liquid ethane and stored under liquid nitrogen before data collection.

TEM data collection
Room temperature MicroED data was acquired on either a Talos F200C TEM operating at 200 kV and equipped with a Direct Electron Apollo detector, or a ThermoFisher Scientific Spectra 300 TEM operating at 300 kV and equipped with a Ceta-D scintillator-based detector. Cryo KLVFFA data was collected using the Talos F200C. For either TEM, LCs were loaded in a single-tilt sample holder at room temperature. First, using SerialEM, a low magnification atlas of the sample was recorded to identify crystal positions and distinguish intact (wet) and breached (dry) regions of the grid. At a selection of microcrystal positions, series of images either in imaging mode, or in diffraction mode with a 100 μm selected area aperture projecting an area of approximately 2.5 μm diameter on the conjugate image plane, were recorded to assess the susceptibility of the microcrystal and surrounding liquid environment to radiolytic damage. The imaging mode settings used here were configured to mimic the incident flux density of the beam in diffraction mode, using the same condenser lens currents in either configuration. For all measurements, low flux conditions were configured using spot size 11 with a 50 μm condenser aperture, producing a beam flux density of 0.01 $e^-/Å^2s$ on the Talos F200C and 0.0048 $e^-/Å^2s$ on the Spectra 300.

Continuous-rotation electron diffraction tilt series were recorded at selected locations on the sample containing microcrystals embedded in liquid. For the Talos F200C / DE Apollo

configuration, tilt series were recorded with a stage rotation rate of 1°/s, and 60 native (60 Hz) frames summed per final saved frame to give an effective integration time per final frame of 1 s. For the Spectra 300 / Ceta-D setup, tilt series were recorded with a stage rotation rate of 0.5°/s with an integration time of 2 s per frame. Diffraction image stacks from the DE Apollo were saved as MRC files and converted to SMV file format using custom scripts written in MATLAB version 2023b. Diffraction image stacks from the Ceta-D were saved directly in SMV file format by EPU-D data collection software.

For comparative imaging of liquid filled and dry grid cells, liquid cells were prepared using 300 mesh Formvar/carbon TEM grids, filled with 1 µL of diluted d-AVAAGA crystal slurry as described previously. The liquid cells were then loaded into a Simple Origin Model 205 dual-grid cryo-transfer holder and inserted into the TEM. Room temperature TEM imaging was performed on a Talos F200C TEM operating at 200 kV and equipped with a Direct Electron Apollo detector. Using SerialEM, an atlas of the sample was recorded at 155x magnification using spot size 11 and an integration time of 1 s per image. The resulting MRC image stack was stitched to generate an atlas showing a portion of the grid while liquid remained trapped between the carbon films. Following initial imaging, the grid was exposed to vacuum for 24 hours, allowing the cell to completely dry. The sample holder was then re-inserted into the TEM and an atlas of the same area was collected under the same imaging conditions. The wet and dry atlases were compared to assess differences in the appearance of the sample before and after drying.

Data processing and structure determination

From diffraction movies corresponding to individual tilt series wedges on distinct crystals, reflections were indexed and integrated using *XDS*,[1] and intensities scaled and merged with those of other isomorphous crystal in *XSCALE.*[2] For each experiment, as many isomorphous wedges of data were merged as could be without incurring large penalties to merge R factors. If the resolution of the merged dataset where I/σ exceeded 2.0 was finer than 1.2 Å, phases were determined by direct methods using either *SHELXT* or *SHELXD.*[3] These *ab initio* structures were refined using *SHELXL* using the graphical interface *SHELXLE,*[4,5] where electron form factors parameterized as four Gaussian functions were used for atoms of each element present. Strong positive peaks in the difference Fourier map within 2.7 to 3.3 Å of a potential hydrogen bond partner were identified during refinement and modeled as water molecules. In the case of the KLVFFA peptide, *PHASER* was used for molecular replacement,[6] and the structure was refined using *PHENIX.*[7]

Electron energy-loss spectroscopy (EELS) analysis of liquid layer thickness

LC samples containing milli-Q water, or microcrystal slurries of Zn(II)-L-histidine were loaded at room temperature into a TFS Spectra 300 operating at 300 keV and equipped with a Ceta-D, a GIF Continuum energy filter, and a post filter Gatan Continuum CMOS detector. EELS spectra were recorded in TEM mode at 2550x magnification with a parallel beam and a 2.8 µm diameter illuminated area defined by the C2 and C3 condenser lenses. These settings were determined such that the beam matched the size of the energy filter's entrance aperture, and at spot size 9 with a monochromator focus value of -20 produced a beam of flux density ~4.8 $e^-/Å^2s$. No objective aperture was inserted during the measurement, allowing for the assumption of a large collection angle, >100 mrad. DigitalMicrograph (Gatan Inc.) was used to record dual-EELS spectra from the illuminated areas using a dispersion of 0.6 eV/channel, measuring a low-loss

spectrum encompassing an energy-loss range of -120 to ~1110 eV, and a high-loss spectrum encompassing an energy-loss range of ~160 to ~1400 eV. The low-loss exposure time was set to $5x10^{-5}$ seconds per frame, and the high-loss exposure time was set to 10 ms per frame, with a total acquisition time of 10 seconds per spectrum. The average thickness of each illuminated region was computed using the log-ratio method as implemented in DigitalMicrograph after extracting the zero-loss peak and inelastic scattering signal from the plasmon in the low-loss spectra.

Figures

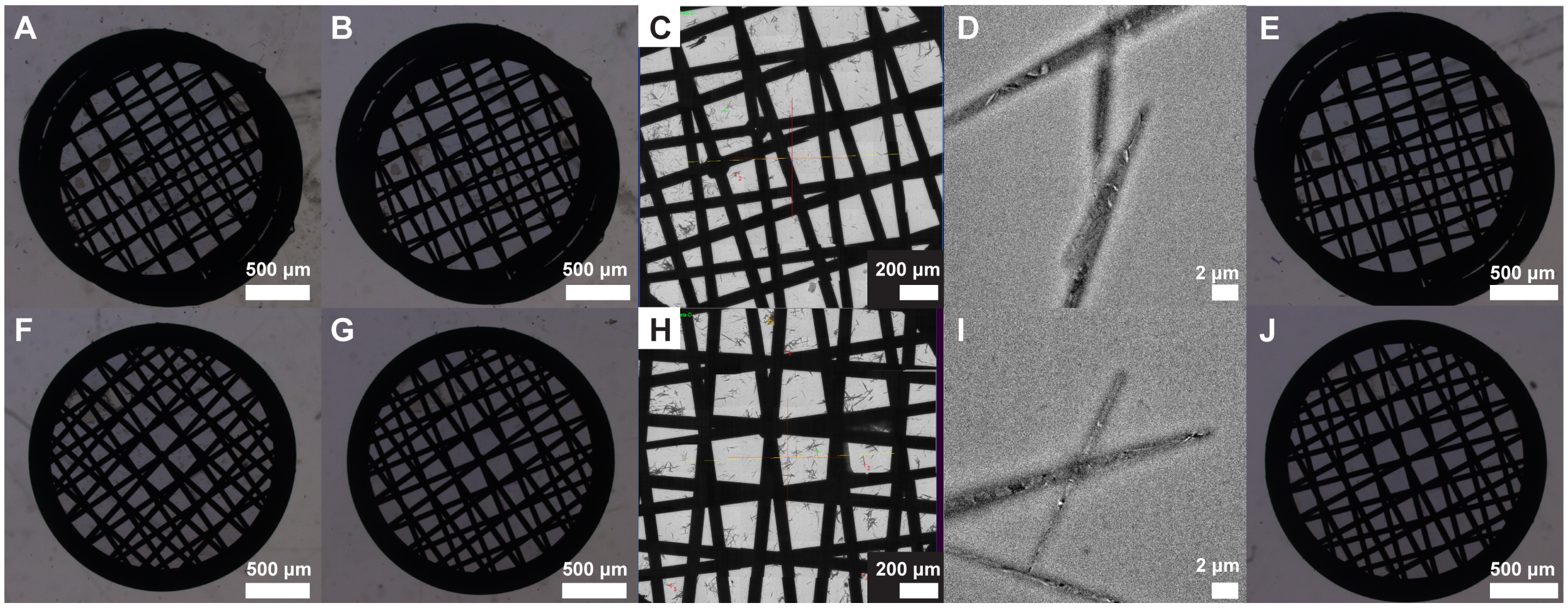

Figure S1. Stability of liquid cells under vacuum with and without grease as sealant. Light microscopy image of liquid cell containing d-AVAAGA microcrystal slurry sealed with high vacuum grease immediately following preparation (A) and after 1 hour in 0.01 torr vacuum chamber (B). TEM atlas (C) and TEM image of a representative hydrated microcrystal (D), and light microscopy image of the same liquid cell following extraction from the TEM (E). Light microscopy image of liquid cell containing d-AVAAGA microcrystal slurry prepared without high vacuum grease immediately following preparation (F) and after 1 hours in 0.01 torr vacuum chamber (G). TEM atlas (H) and TEM image of a representative hydrated microcrystal (I), and light microscopy image of the same liquid cell following extraction from the TEM (J).

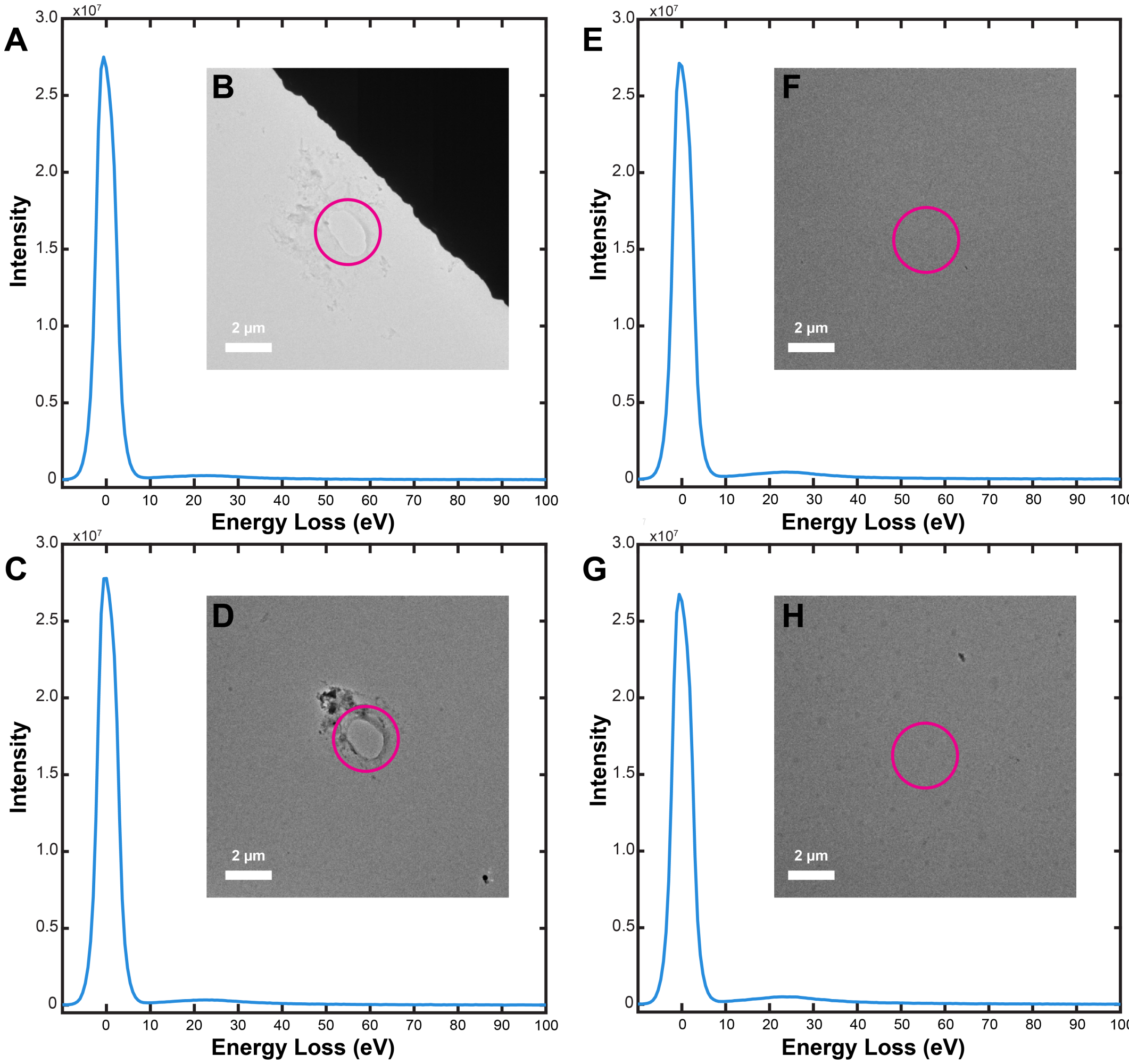

Figure S2. EELS measurements of dry and liquid regions in a water LC. EELS low-loss spectra (A,C) recorded from apparently dry regions of an LC sample prepared with only water inside, and corresponding TEM images (B,D), where the magenta circle indicates the region the EELS spectrum was recorded from. These are compared to EELS low-loss spectra (E,G) and corresponding TEM images (F,H) from apparently water-filled regions in the proximity (no more than one grid square away) of the dry area measured in A-D.

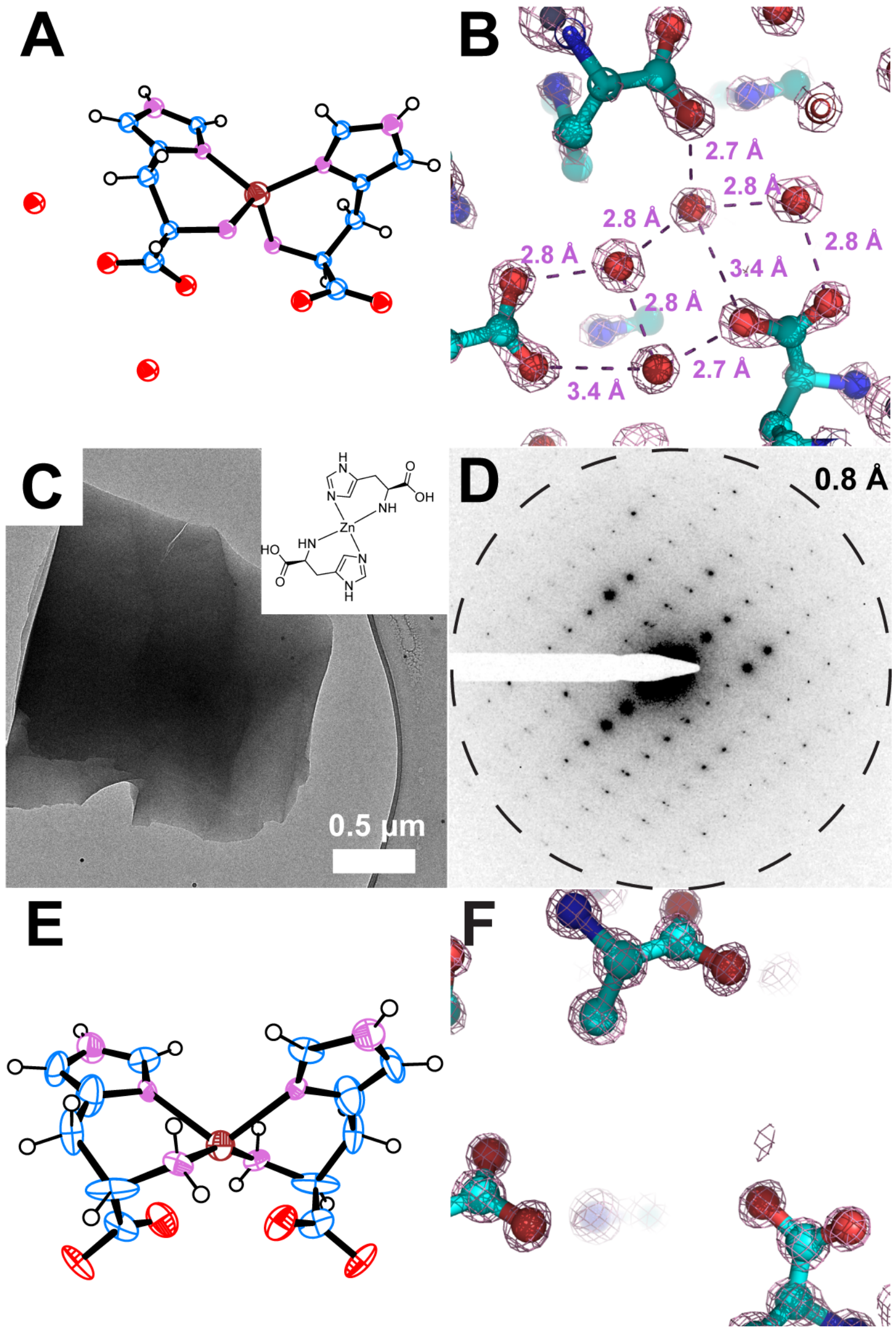

Figure S3. Single-crystal XRD and dry room temperature structures of Zn(II)-histidine. ORTEP diagram of solvated Zn(II)-histidine structure determined by single-crystal XRD at 100 K to 1.4 Å resolution (A), and view of model overlaid with F_{obs} electron density map showing sites of two water molecules (B). TEM image of dry Zn(II)-histidine microcrystal at room temperature (C), and diffraction pattern from the same crystal (D). ORTEP diagram of the dry-RT Zn(II)-histidine structure determined by MicroED to 0.8 Å resolution (E), and equivalent view of the model overlaid with F_{obs} electrostatic potential map as shown for the XRD structure in panel B (F).

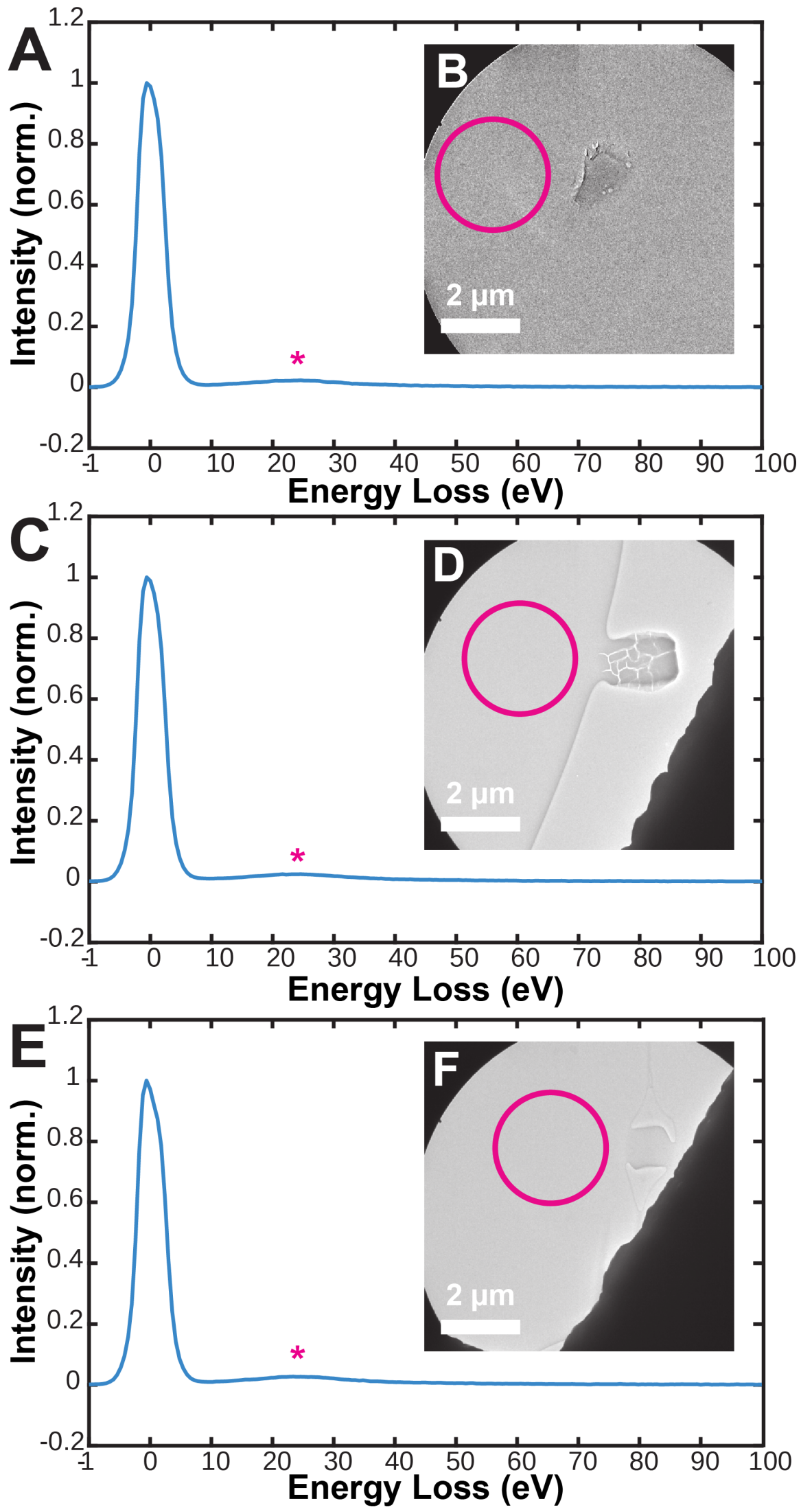

Figure S4. EELS measurements of liquid regions adjacent to microcrystals in a Zn(II)-L-histidine LC. EELS low-loss spectra recorded from the 2.8 μm diameter regions adjacent to Zn(II)-L-Histidine microcrystals that appear to be immersed in liquid (A,C,E), and corresponding TEM images of those microcrystals, where the magenta circle in each case indicates the region where the EELS spectrum was recorded from (B,D,F). Magenta asterisks in the low-loss EELS spectra indicate the rough maximum of the plasmon peak adjacent to the zero-loss peak.

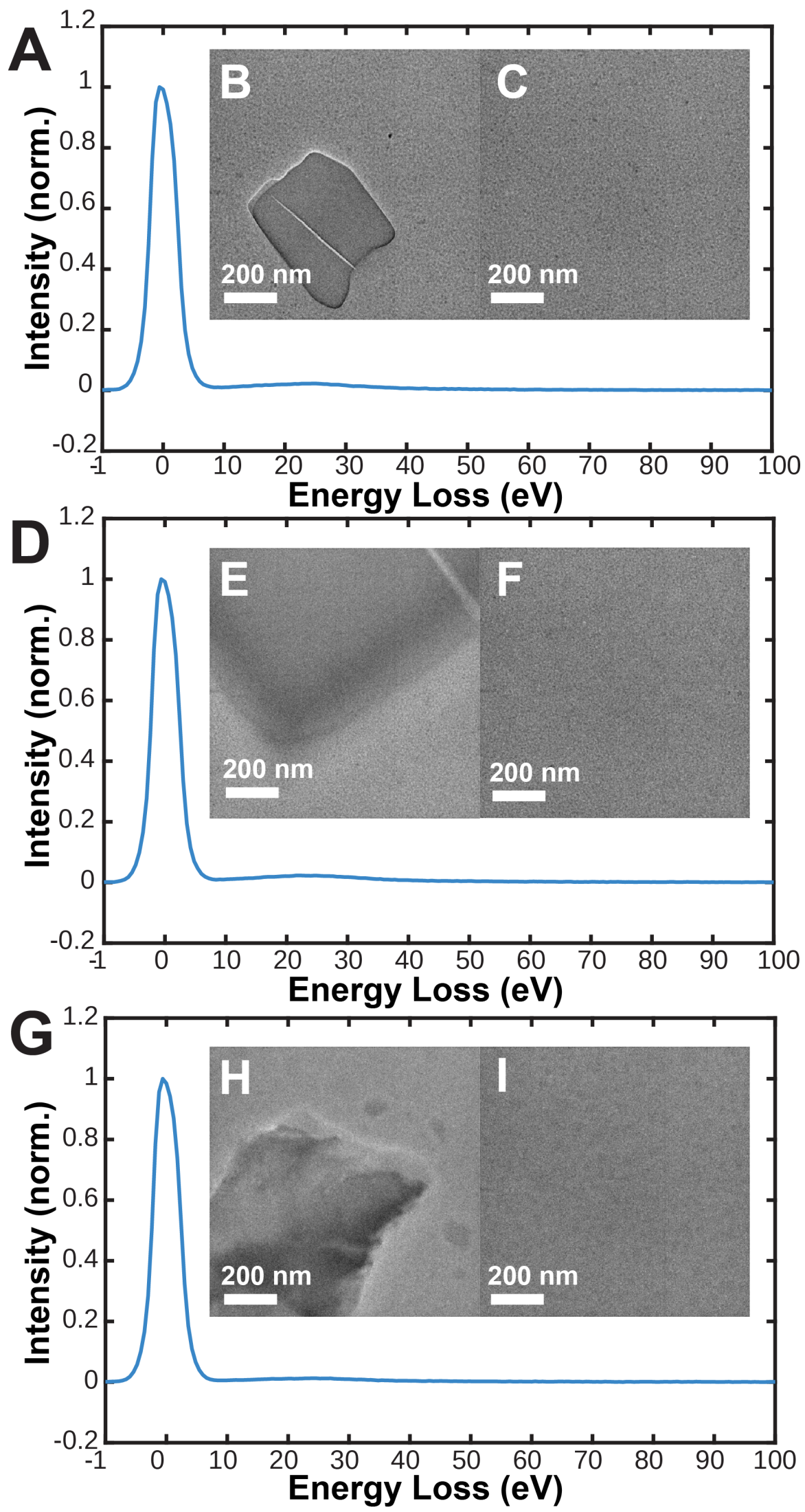

Figure S5. EELS measurements of regions adjacent to microcrystals in a dried-out Zn(II)-L-histidine LC sample. EELS low-loss spectra recorded from 2.8 μm diameter regions adjacent to Zn(II)-L-Histidine microcrystals mounted in a carbon sandwich LC that was allowed to fully dry under ambient conditions over a four-day period (A.D.G), corresponding TEM images of those microcrystals (B,E,H), and TEM images of the empty regions adjacent to each microcrystal where an EELS spectrum was recorded from (C,F,I).

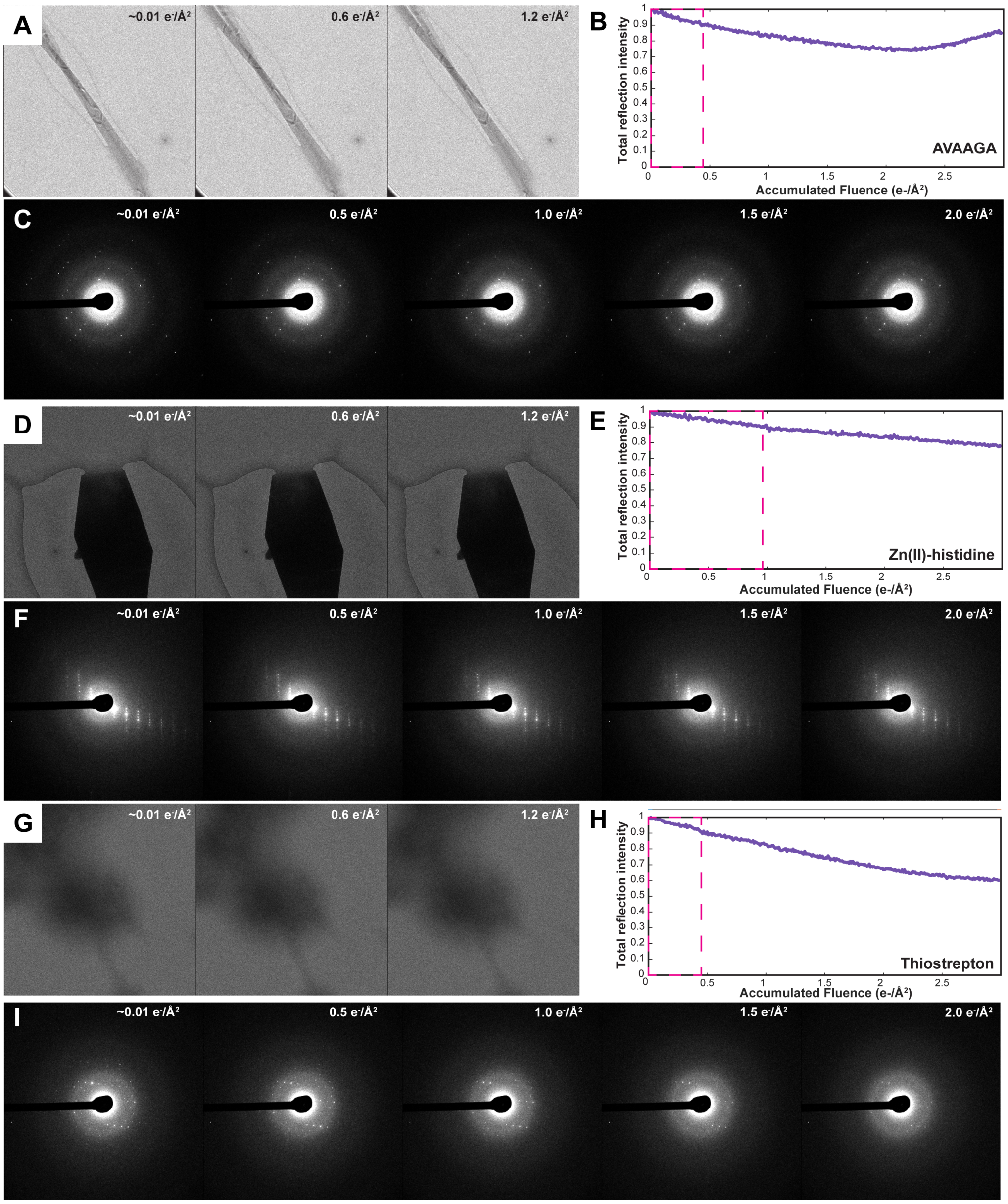

Figure S6. Establishing dose budgets for beam-sensitive LC microcrystal samples. Frames from a TEM imaging dose series acquired on static hydrated crystals of d-AVAAGA following 0.01, 0.6, and 1.2 e-/Å^2 accumulated fluence (A). From a diffraction mode dose series acquired on a different static hydrated d-AVAAGA crystal in the same LC grid, plot of the net intensity of the 20% brightest reflections detected normalized to its maximum value as a function of fluence, where the magenta dashed box highlights the fluence domain where net intensity exceeds 90% of its original value (B), and frames from this diffraction series following 0.01, 0.5, 1.0, 1.5, and 2.0 e-/Å^2 accumulated fluence (C). The same are shown for crystals of Zn(II)-histidine (D-F) and thiostrepton (G-I). All measurements were acquired at room temperature at 200 keV, with in incident flux density of 0.01 e-/Å^{2}s.

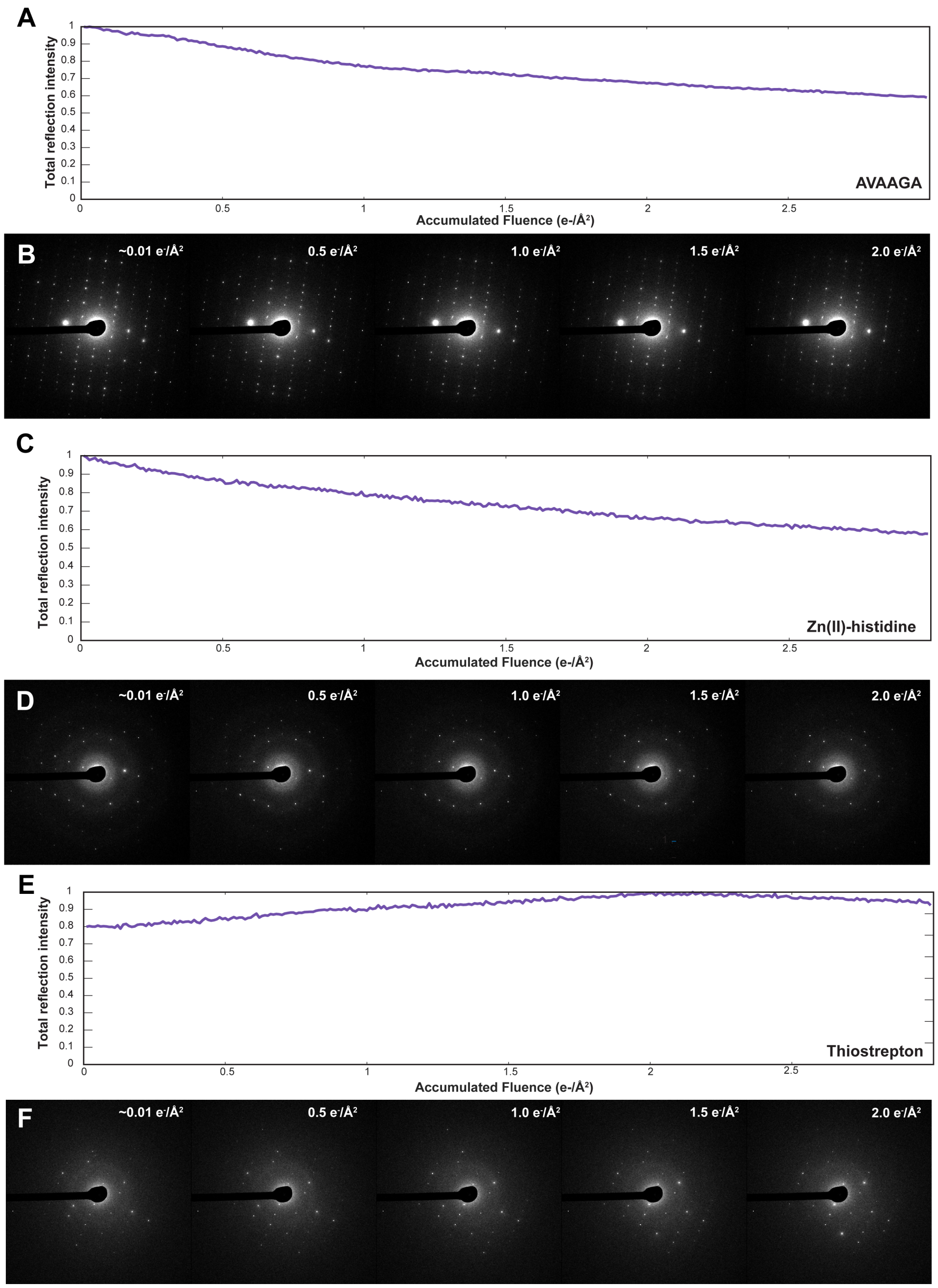

Figure S7. Dose tolerance for dry microcrystals at cryogenic temperature. From a diffraction mode dose series acquired on a static dry d-AVAAGA crystal at 100 K, plot of the net intensity of the 20% brightest reflections detected normalized to its maximum value as a function of fluence (A), and frames from this diffraction series following 0.01, 0.5, 1.0, 1.5, and 2.0 e-/Å^2 accumulated fluence (B). The same are shown for crystals of Zn(II)-histidine (C-D) and thiostrepton (E-F). All measurements were acquired at room temperature at 200 keV, with an incident flux density of 0.01 e-/Å^{2}s.

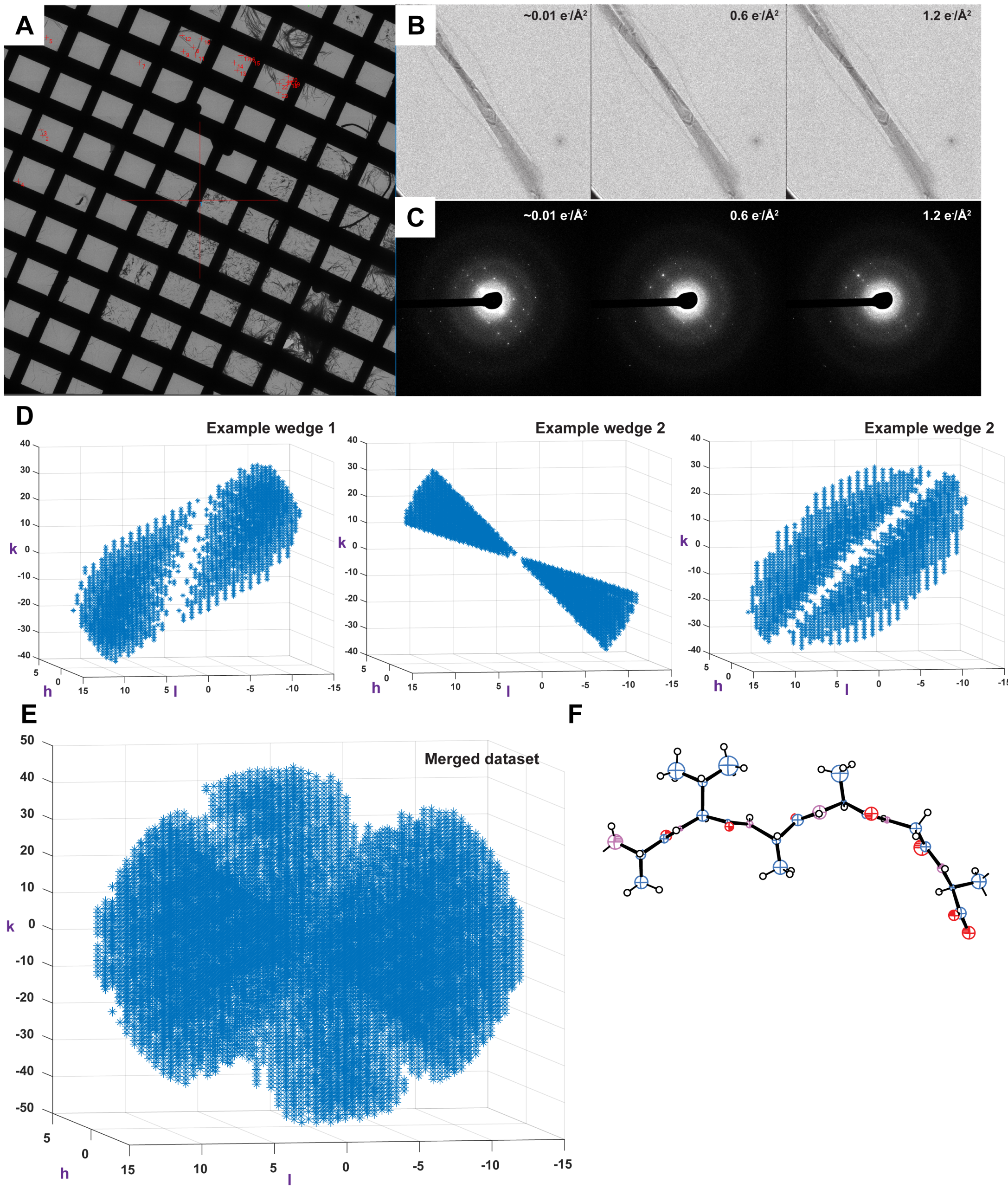

Figure S8. Dose-budget approach applied to LC-MicroED structure determination of d-AVAAGA. Atlas of a room-temperature, hydrated TEM grid of d-AVAAGA peptide crystals, where red markers indicate positions short diffraction tilt series were acquired (A). Selected frames from an imaging-mode dose series on one representative static solvated d-AVAAGA crystal at room temperature (B), and from a diffraction-mode dose series on another (C), informing on the permissible electron dose that can be delivered while maintaining solvation and diffraction quality of the crystal respectively. Based on this information, many short wedges of MicroED data limiting fluence to 0.3 $e^{-}/Å^{2}$ were recorded. Three such partial datasets are shown in D, while 10 total were merged (E) to yield an atomic resolution structure (F).

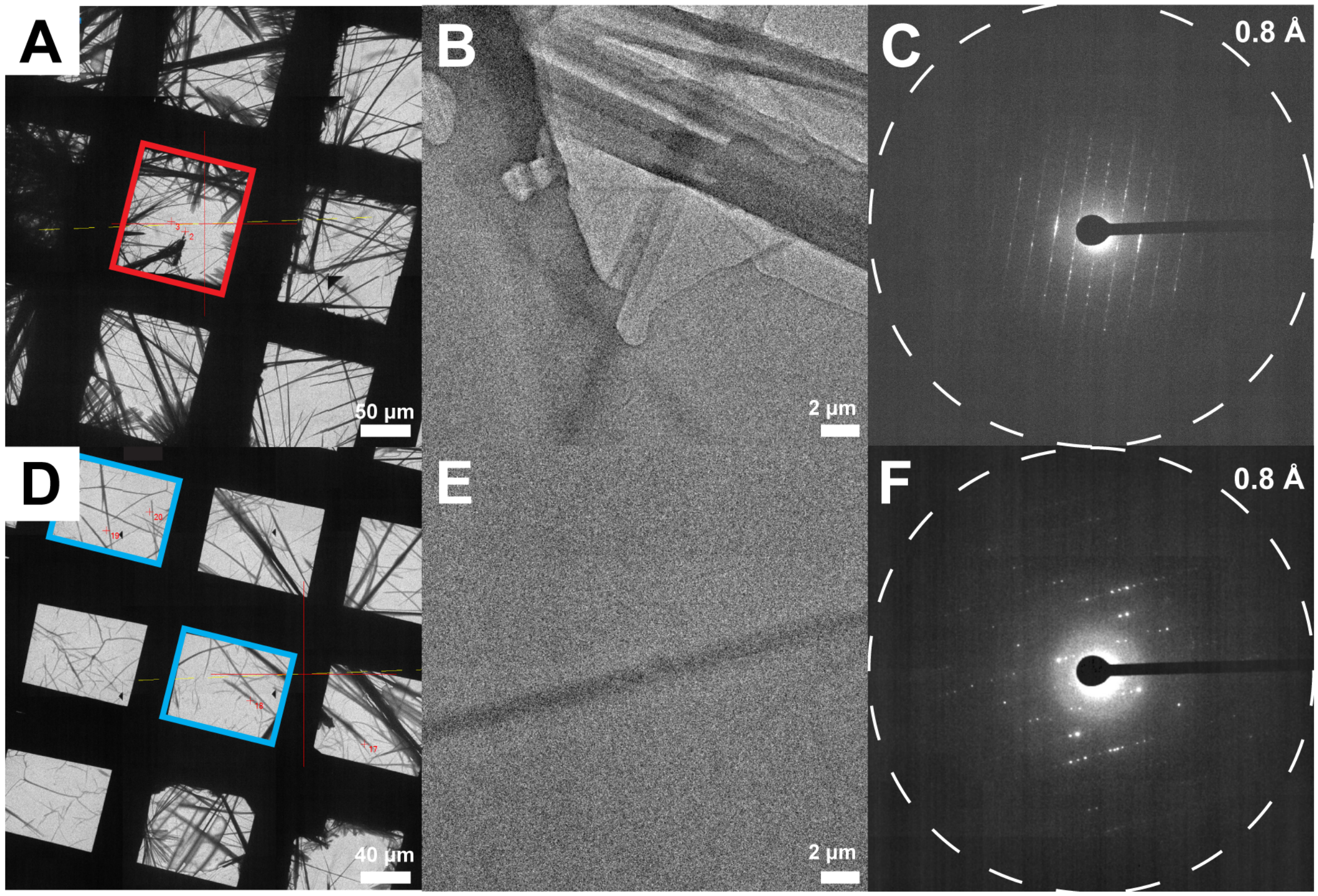

Figure S9. Electron diffraction of dehydrated and hydrated 3PhCOOK crystals. View of TEM atlas acquired from a liquid cell of 3PhCOOK crystals, where a dried-out area of the grid is highlighted with a red box (A). TEM image of the interface between a wet and dry area of the 3PhCOOK LC sample (B), and diffraction pattern from a dry 3PhCOOK microcrystal (C). View of a different region of the same TEM atlas with apparently wet regions of the grid highlighted in blue boxes (D), TEM image of a 3PhCOOK microcrystal suspended in liquid within the LC (E), and diffraction pattern from a representative wet microcrystal (F).

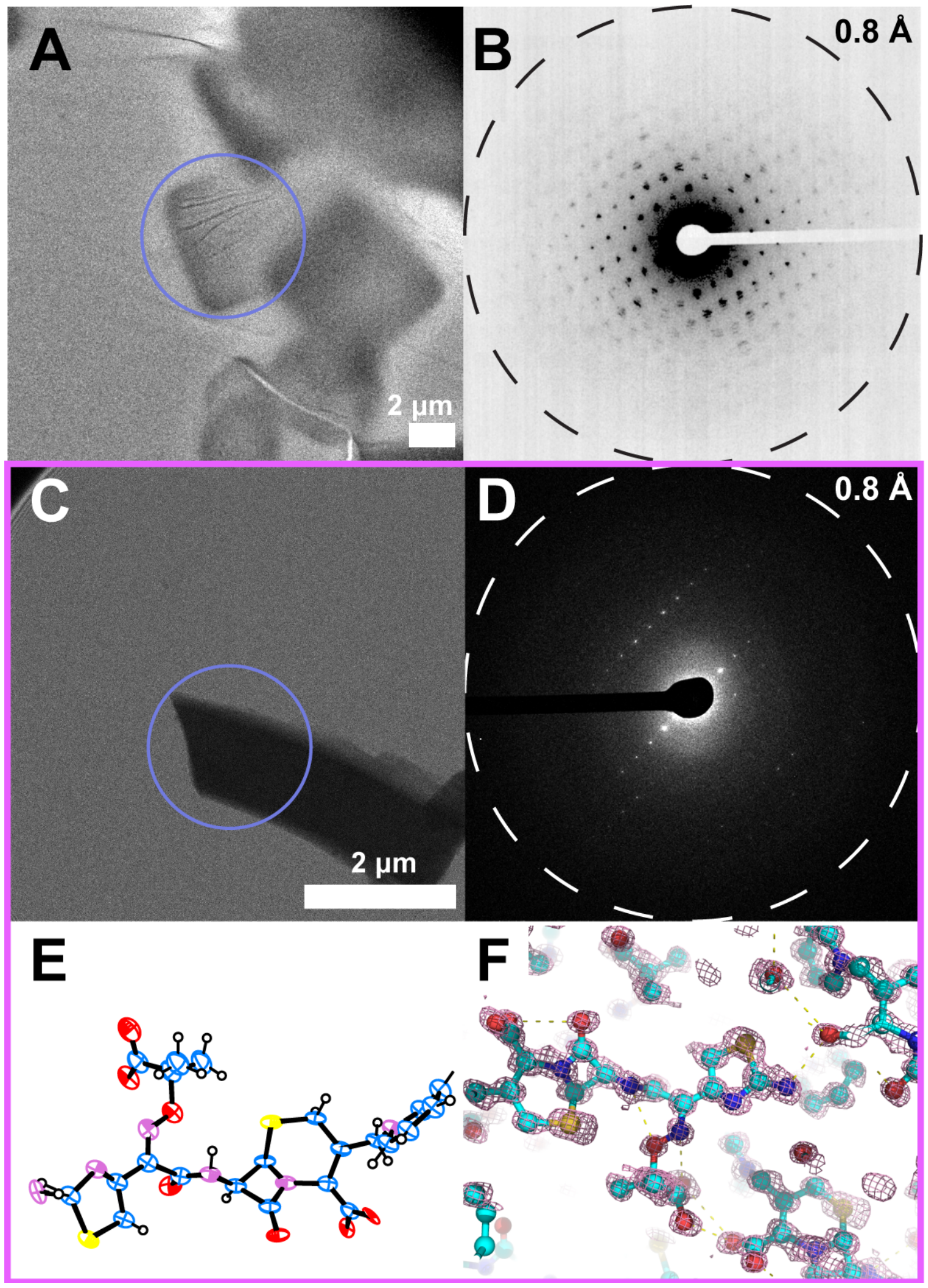

Figure S10. MicroED of dry pentahydrate ceftazidime and dry monohydrate ceftazidime. TEM image acquired at room temperature of ceftazidime pentahydrate microcrystals prepared dry on a grid and inserted in the TEM (A), and diffraction pattern acquired from the crystal in the circled area showing evidence of disorder or partial lattice collapse (B). TEM image acquired at room temperature of ceftazidime monohydrate microcrystals prepared dry on a grid and inserted in the TEM (C), and diffraction pattern acquired from the crystal in the circled area (D). ORTEP diagram of MicroED structure solution from the microcrystal in panel C (E), and model overlaid with 1.5σ level electrostatic potential map showing crystal packing with no evidence of any ordered water molecules in the lattice (F).

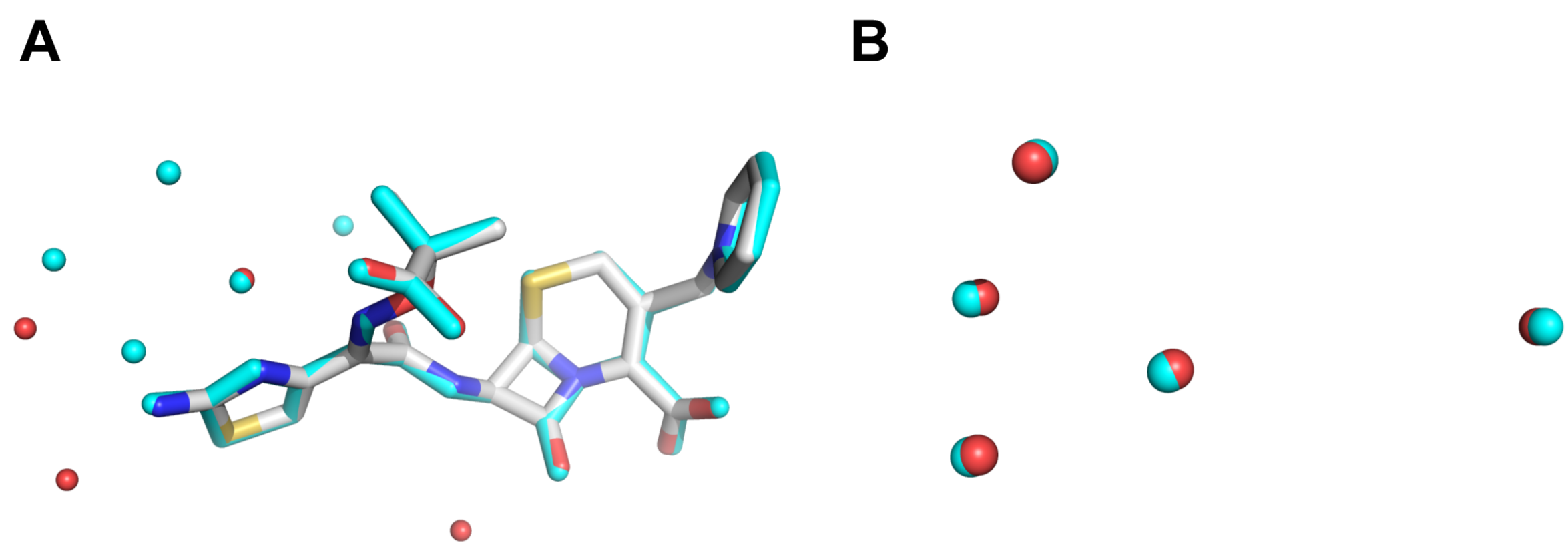

Figure S11. Alignment of ceftazidime pentahydrate structures from single-crystal XRD and liquid cell MicroED. Overlay of asymmetric units of a known ceftazidime pentahydrate structure determined by single-crystal XRD (cyan coordinates) and ceftazidime pentahydrate structure determined by liquid cell MicroED (white, blue, red, and yellow coordinates) (A). Enhanced view of the five coordinated water molecules with symmetry applied to the liquid cell MicroED structure showing agreement in position for each between the XRD (cyan) and LC-MicroED (red) structures (B).

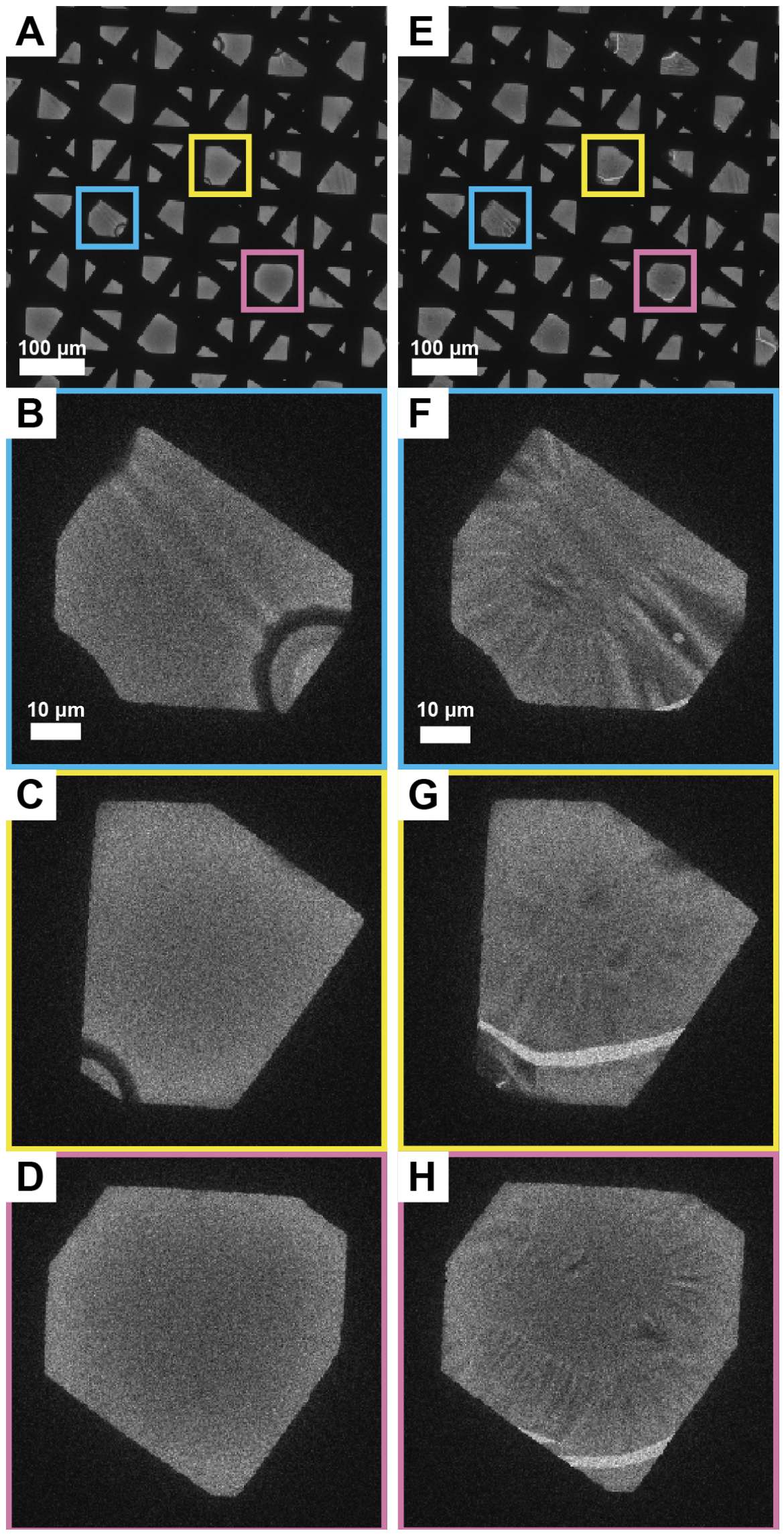

Figure S12. Comparison of liquid filled and subsequently dried cells containing a d-AVAAGA crystal slurry. (A-D) Low-magnification montage of a liquid-cell grid, filled with a slurry of d-AVAAGA crystals and imaged with liquid retained between the carbon films. (E-H) Montage of the same grid region following 24 h of drying under vacuum. Colored boxes indicate corresponding regions of interest in the wet (B-D) and dry (F-H) montages, with enlarged insets depicted below the corresponding montage. The matched colored regions highlight differences in appearance of crystal outlines and their surroundings before and after drying in vacuum. Scale bars: 100 µm in full montages, 10 µm in the enlarged views.

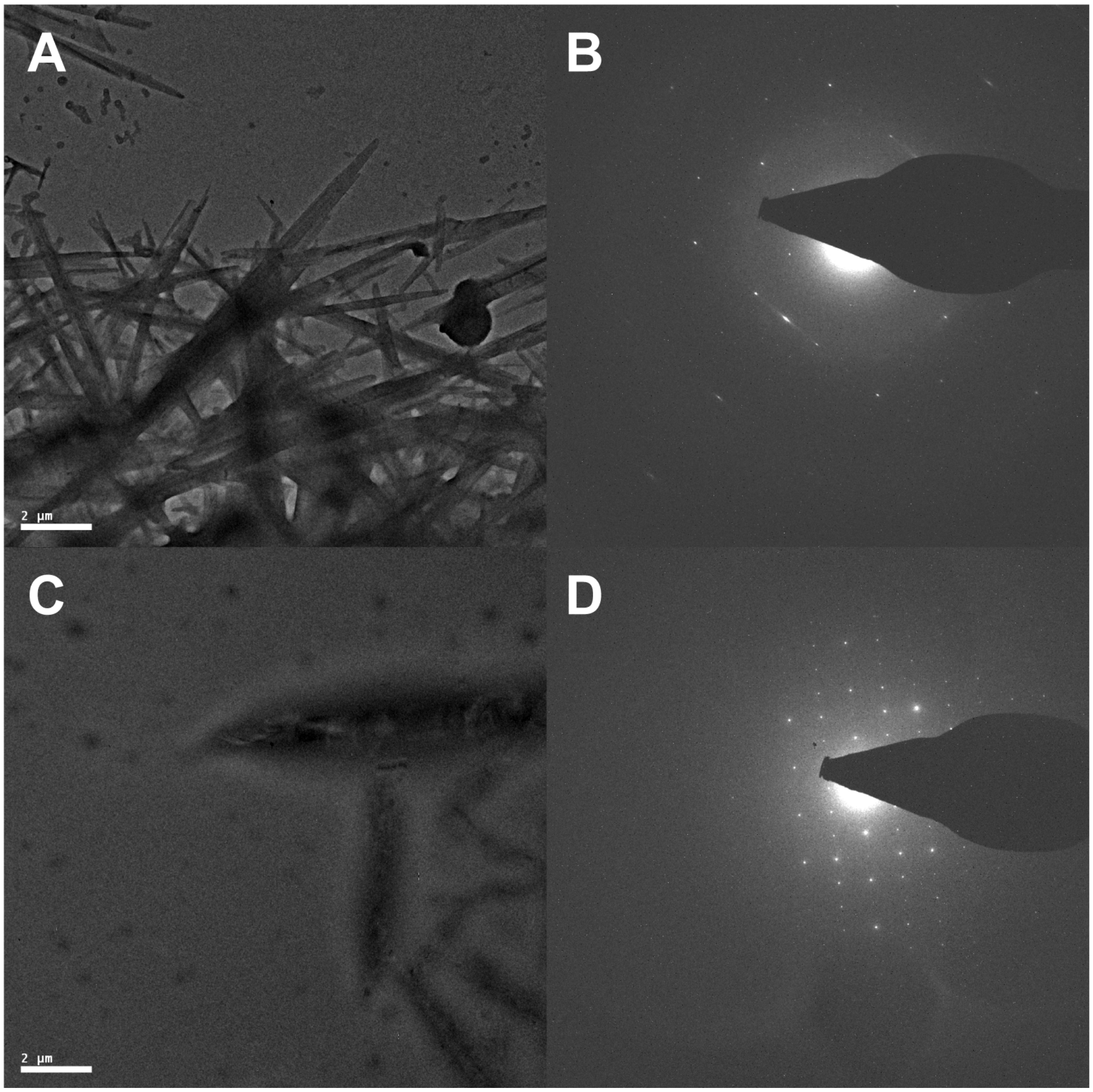

Figure S13. Diffraction from KLVFFA microcrystals dry at room temperature, and in liquid cell at room temperature. TEM image of dry KLVFFA microcrystal at room temperature (A) and diffraction pattern (B). TEM image of hydrated KLVFFA microcrystal at room temperature in a liquid cell (C) and diffraction pattern (D). All images shown were acquired at 120 keV.

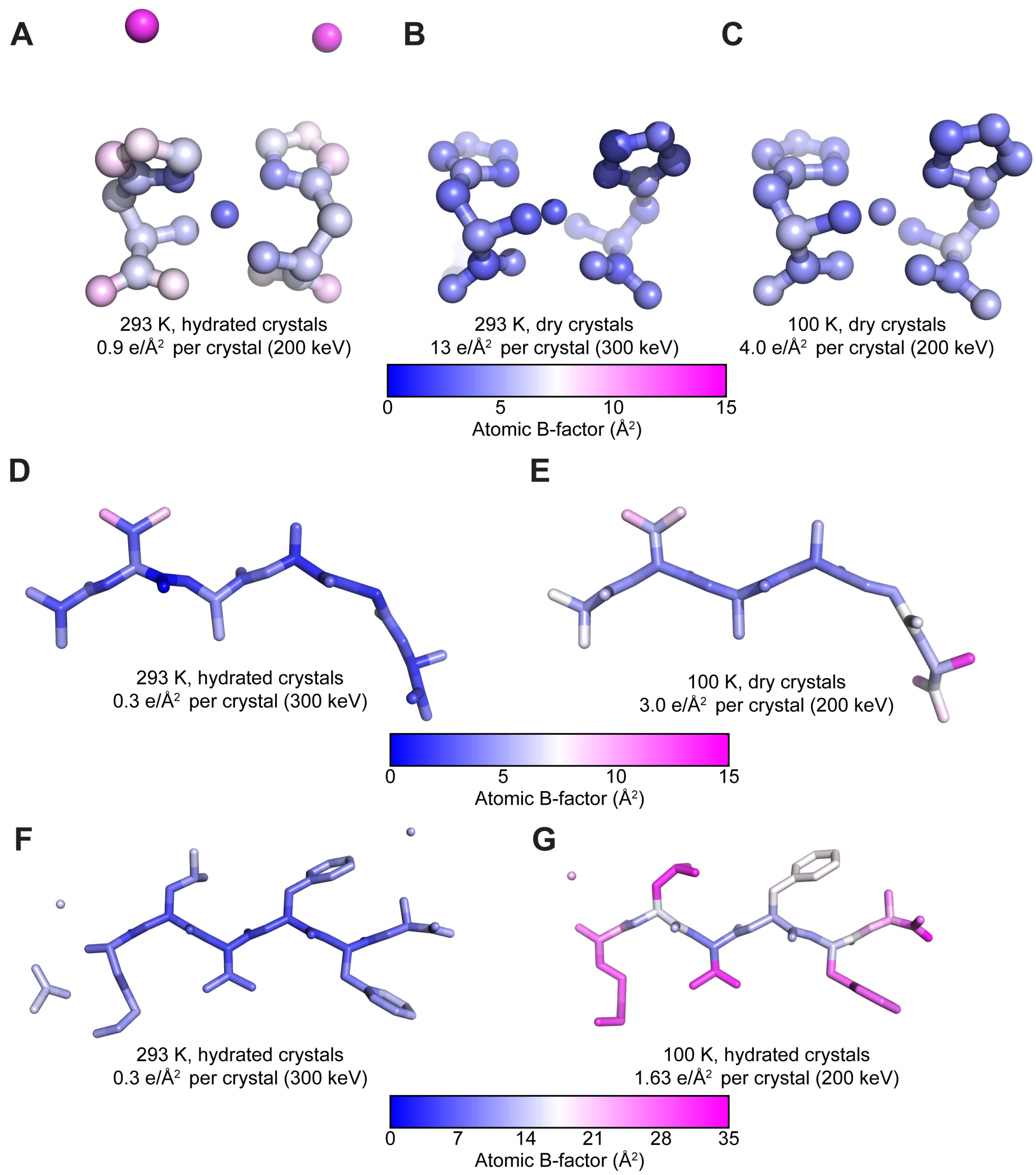

Figure S14. B-factors in LC-MicroED structures of Zn(II)-histidine, d-AVAAGA, and KLVFFA compared to conventional MicroED structures. LC-MicroED structure of Zn(II)-D-histidine (A), dry room-temperature MicroED structure of Zn(II)-L-histidine (B), and dry 100 K MicroED structure (C) of Zn(II)-L-histidine all determined to 0.8 Å resolution, with atomic coordinates colored by B-factor. LC-MicroED structure of d-AVAAGA determined to 0.8 Å resolution (D), and conventional dry 100 K MicroED structure of d-AVAAGA determined to 0.9 Å resolution , with atomic coordinates colored by B-factor (A). LC-MicroED structure of KLVFFA determined to 1.0 Å resolution (F), and conventional frozen-hydrated 100 K MicroED structure of KLVFFA determined to 1.5 Å resolution (G), with atomic coordinates colored by B-factor. B-factors across all structures determined from LC samples range between 0 and 15 Å^2.

Site	Fraction of inelastic mean-free-path	Estimated thickness (nm)
**1**	0.09	25.2
**2**	0.09	25.2
**3**	0.10	28
**4**	0.09	25.2
**5**	0.10	28
**6**	0.09	25.2
**7**	0.09	25.2
**8**	0.10	28
**9**	0.10	28
**10**	0.10	28
**Dry 1**	0.07	19.6
**Dry 2**	0.05	14
**Dry 3**	0.08	22.4

Table S1. Average thickness of 2.8 μm diameter areas of an LC sample containing only water, as measured by EELS using the log-ratio method on low-loss zero-loss peak and inelastic plasmon peak measurements. The first ten randomly selected sites were all suspected to contain liquid water based on TEM contrast relative to visibly dry regions of the sample, while the final three sites were all from regions that appeared visibly dry. Calculations assume a simple model where the sample inelastic mean-free-path of 300 keV electrons is that of water, 280 nm. The second column reports the average number of inelastic mean free paths (τ/λ) in the illuminated area, calculated by the log-ratio method taking $-\ln[(I_{zlp}/I_{spectrum})]$ where I_{zlp} is the integrated intensity of the zero loss peak and $I_{spectrum}$ is the integrated intensity of the whole measured spectrum. The difference in average thickness measured between wet and dry measured regions is consistent with a 7.9 nm thick layer of liquid water.

	Zn(II)-L-Histidine (XRD)	Zn(II)-L-Histidine (MicroED, RT-dry)	Zn(II)-D-Histidine (MicroED, RT-liquid cell)	Zn(II)-L-Histidine (MicroED, 100K-dry)
**CCDC number**	N/A	2481287	2481286	2521667
**Data Collection and Processing**				
No. crystals merged	1	1	1	1
Incident beam Wavelength (Å)	1.74	0.0197	0.0251	0.0251
Temperature (K)	100	293	293	100
Resolution (Å)	5.44 – 1.4 (1.5 – 1.4)	6.82 – 0.8 (0.9 – 0.8)	7.43 – 0.8 (0.9 – 0.8)	6.63 – 0.8 (0.9 – 0.8)
Space Group	$P4_32_12$	$P4_32_12$	$P4_12_12$	$P4_32_12$
a, *b*, *c* (Å)	7.59, 7.59, 31.16	7.02, 7.02, 29.07	7.28, 7.28, 29.71	6.83, 6.83, 28.25
α, *β*, *γ* (°)	90, 90, 90	90, 90, 90	90, 90, 90	90, 90, 90
# total reflections	522 (68)	5385 (1430)	5038 (1487)	6881 (2037)
# unique reflections	286 (46)	888 (245)	961 (275)	729 (201)
R_{merge} (%)	2.3 (2.2)	16.3 (35.1)	18.3 (44.8)	19.0 (81.4)
CC1/2 (%)	99.8 (99.6)	99.0 (91.0)	98.5 (22.8)	99.5 (85.2)
<I/σI>	29.43 (28.10)	7.76 (3.92)	5.08 (2.58)	8.46 (2.94)
Completeness (%)	82.7 (66.7)	93.4 (90.4)	92.9 (94.2)	83.4 (83.1)
**Phasing**				
Software	*SHELXT*	*SHELXD*	*SHELXD*	*SHELXT*
**Refinement**				
Resolution (Å)	5.44 – 1.4	6.82 – 0.8 Å	7.43 – 0.8 Å	6.63 – 0.8 Å
R_1 (%)	4.60	15.65	23.99	13.21
wR_2 (%)	11.25	43.81	58.20	35.48
Goodness of fit	1.096	1.682	1.808	1.294

Table S2. Statistics of crystallographic data reduction and refinement for structures of Zn(II)-histidine determined by single crystal XRD (100 K, hydrated), room temperature MicroED on dry crystals, room temperature MicroED in a liquid cell, and cryogenic (100 K) MicroED on dry crystals.

Site	Fraction of inelastic mean-free-path	Estimated thickness (nm)
**Wet 1**	0.13	36.4
**Wet 2**	0.13	36.4
**Wet 3**	0.14	39.2
**Dry 1**	0.11	30.8
**Dry 2**	0.12	33.6
**Dry 3**	0.07	19.6

Table S3. Average thickness of 2.8 μm diameter areas of an LC sample containing Zn(II)-L-histidine microcrystals (Wet 1, Wet 2, and Wet 3), as measured by EELS using the log-ratio method on low-loss zero-loss peak and inelastic plasmon peak measurements, and a desiccated Zn(II)-L-histidine LC (Dry 1, Dry 2, and Dry 3). A simple model for the sample here assumes an inelastic mean-free-path of 300 keV electrons in water, 280 nm. The second column reports the average number of inelastic mean free paths (τ/λ) in the illuminated area, calculated by the log-ratio method taking $-\ln[(I_{zlp}/I_{spectrum})]$ where I_{zlp} is the integrated intensity of the zero loss peak and $I_{spectrum}$ is the integrated intensity of the whole measured spectrum. The difference in average thickness measured between wet and dry measured regions in the proximity of Zn(II)-L-histidine crystals is consistent with a 9.3 nm thick layer of liquid water.

**Sample**	Permissible fluence ($e^-/Å^2$)	Accelerating voltage (kV)
**Zn(II)-histidine**	0.97	200
**3PhCOOK**	0.22	300
**Ceftazidime pentahydrate**	0.39	300
**d-AVAAGA**	0.45	200
**KLVFFA**	0.27	300

Table S4. Permissible beam fluence for room temperature hydrated samples. The average fluence at which finer than 1.2 Å diffraction is lost in LC samples is tabulated. Some samples were measured at an accelerating voltage of 200 kV, and some were measured at 300 kV.

	3PhCOO-K
**CCDC number**	2481288
**Data Collection and Processing**	
No. crystals merged	9
Incident beam Wavelength (Å)	0.0197
Temperature (K)	293
Resolution (Å)	12.32 – 1.0 (1.1 – 1.0)
Space Group	$P\bar{1}$
a, *b*, *c* (Å)	8.98, 15.01, 25.49
α, *β*, *γ* (°)	98.610, 100.988, 96.456
# total reflections	21820 (5371)
# unique reflections	4381 (1054)
R_{merge} (%)	20.0 (134.6)
CC1/2 (%)	99.5 (40.3)
<I/σI>	4.51 (1.44)
Completeness (%)	63.5 (62.3)
**Phasing**	
Software	*SHELXT*
**Refinement**	
Resolution (Å)	12.32 – 1.0
R_1 (%)	13.37
wR_2 (%)	39.50
Goodness of fit	1.250

Table S5. Statistics of crystallographic data reduction and refinement for structures of triphenyl acetic acid salt solvates determined by MicroED from room temperature liquid cells.

	Ceftazidime pentahydrate (RT-liquid cell)	Ceftazidime monohydrate (RT-dry)
**CCDC number**	2481289	2481290
**Data Collection and Processing**		
No. crystals merged	8	1
Incident beam Wavelength (Å)	0.0197	0.0251
Temperature (K)	293	293
Resolution (Å)	8.58 – 0.8 (0.9 – 0.8)	8.86 – 0.8 (0.9 – 0.8)
Space Group	$P2_12_12_1$	$P2_12_12_1$
a, *b*, *c* (Å)	8.92, 10.29, 31.21	9.46, 10.07, 25.44
α, *β*, *γ* (°)	90, 90, 90	90, 90, 90
# total reflections	28765 (7725)	11239 (3185)
# unique reflections	2726 (782)	2403 (695)
R_{merge} (%)	28.5 (228.5)	16.6 (47.1)
CC1/2 (%)	97.2 (2.6)	99.3 (10.4)
<I/σI>	5.06 (1.36)	5.61 (2.51)
Completeness (%)	81.9 (81.8)	84.2 (86.0)
**Phasing**		
Software	*SHELXD*	*SHELXD*
**Refinement**		
Resolution (Å)	8.58 – 0.8	8.86 – 0.8
R_1 (%)	15.19	25.10
wR_2 (%)	41.36	56.16
Goodness of fit	1.167	1.756

Table S6. Statistics of crystallographic data reduction and refinement for structures of ceftazidime determined at room temperature dry and in liquid cells by MicroED.

	d-AVAAGA (RT-liquid cell)	d-AVAAGA (100K-dry)
**CCDC Number**	2481974	2521666
**Data Collection and Processing**		
No. crystals merged	10	3
Incident beam Wavelength (Å)	0.0251	0.0251
Temperature (K)	293	100
Resolution (Å)	6.57 – 0.8 (0.9 – 0.8)	10.76 s– 0.8 (0.9 – 0.8)
Space Group	$P2_12_12_1$	$P2_12_12_1$
a, *b*, *c* (Å)	4.74, 11.64, 39.42	4.66, 11.20, 38.62
α, β, γ (°)	90, 90, 90	90, 90, 90
# total reflections	26257 (7290)	19927 (5101)
# unique reflections	2418 (676)	2201 (610)
R_{merge} (%)	24.7 (55.7)	22.3 (181.7)
CC1/2 (%)	98.3 (47.0)	99.3 (32.7)
$\langle I/\sigma I \rangle$	6.90 (3.41)	5.26 (1.01)
Completeness (%)	92.1 (91.4)	90.2 (89.7)
**Phasing**		
Software	*SHELXD*	*SHELXD*
**Refinement**		
Resolution (Å)	6.57 – 0.8	10.76 – 0.8
R_1 (%)	25.95	19.04
wR_2 (%)	56.10	45.97
Goodness of fit	2.073	1.389

Table S7. Statistics of crystallographic data reduction and refinement for MicroED structure of d-AVAAGA determined at room temperature in liquid cell, and MicroED structure of d-AVAAGA determined from dry crystals at 100 K.

	KLVFFA (RT-liquid cell)	KLVFFA (cryo)
**PDB number**	9Q2V	11ZN
**Data Collection and Processing**		
No. crystals merged	6	8
Incident beam wavelength (Å)	0.0197	0.0251
Temperature (K)	293	100
Resolution (Å)	11.0 – 1.0 (1.1 - 1.0)	12.02 – 1.5 (1.6 – 1.5)
Space Group	$P2_12_12_1$	$P2_12_12_1$
a, *b*, *c* (Å)	9.68, 12.94, 41.82	9.74, 12.54, 42.26
α, *β*, *γ* (°)	90, 90, 90	90, 90, 90
# total reflections	39087 (9899)	17420 (2764)
# unique reflections	2452 (592)	778 (141)
R_{merge} (%)	28.8 (288.3)	25.7 (66.4)
CC1/2 (%)	99.7 (33.5)	98.6 (90.7)
<I/σI>	4.94 (1.45)	7.35 (3.65)
Completeness (%)	78.1 (79.1)	79.5 (81.5)
**Phasing**		
Software	*PHASER*	*PHASER*
Search model PDB ID	2Y29	2Y29
**Refinement**		
Resolution (Å)	11.0 – 1.0	12.02 – 1.5
R_{work} (%)	28.56	30.88
R_{free} (%)	31.01	33.91

Table S8. Statistics of crystallographic data reduction and refinement for MicroED structure of KLVFFA determined at room temperature in liquid cell.

CheckCIF/Platon report for MicroED structures
Included are responses to A and B level alerts

Zn(II)-histidine (LC-MicroED)
A-level alerts

PLAT082_ALERT_2_A High R1 Value .... 0.24 Report
PLAT084_ALERT_3_A High wR2 Value (i.e. > 0.25) .... 0.58 Report

Author response: The data comes from electron diffraction measurement collected at room temperature for crystals suspended in mother liquor. Some inaccuracies in integration due both to distortions/sources of inaccuracy present in ED data collection, and due to meaurement of crystals in a liquid medium, are expected here.

B-level alerts

PLAT306_ALERT_2_B Isolated Oxygen Atom (H-atoms Missing ?) .... O10 Check

Author response: Clear evidence of H-atoms in the electrostatic potential map attached to coordinated water molecules was not detected, so hydrogen atoms were not modeled on water molecules.

PLAT341_ALERT_3_B Low Bond Precision on C-C Bonds .... 0.0325 Ang.
PLAT430_ALERT_2_B Short Inter D...A Contact O10 ..O11 . 2.78 Ang.
1/2-y,1/2+x,1/4+z = 2_555 Check
PLAT430_ALERT_2_B Short Inter D...A Contact O10 ..O11 . 2.83 Ang.
1+y,1+x,-z = 7_665 Check

Author response: The missing wedge of data in electron diffraction measurements often limits data completeness in certain regions of reciprocal space. This is likely to reduce precision of some bond lengths.

```
PLAT911_ALERT_3_B Missing FCF Refl Between Thmin & STh/L=  0.600       69 Report
         1 1 0,  2 2 0,  2 3 0,  3 3 0,  3 4 0,  4 4 0,
         2 5 0,  3 5 0,  4 5 0,  5 5 0,  4 6 0,  5 6 0,
         6 6 0,  4 7 0,  5 7 0,  1 1 1,  2 2 1,  2 3 1,
         3 3 1,  3 4 1,  4 4 1,  4 5 1,  5 5 1,  4 6 1,
         5 6 1,  6 6 1,  5 7 1,  2 2 2,  3 3 2,  3 4 2,
           (  39 More Missing: see the .ckf listing file)
```

Author response: The missing wedge of data in electron diffraction measurements often limits data completeness in certain regions of reciprocal space.

Zn(II)-histidine (dry room-temperature MicroED)

A-level alerts

No A-level alerts

B-level alerts

PLAT029 ALERT 3 B diffrn measured fraction theta full value Low . 0.947 Why?

Author response: The missing wedge of data in electron diffraction measurements often limits data completeness in certain regions of reciprocal space.

PLAT082_ALERT_2_B High R1 Value .... 0.16 Report

PLAT084 ALERT 3 B High wR2 Value (i.e. > 0.25) .... 0.44 Report

Author response: The data comes from electron diffraction measurement collected at room temperature. Some inaccuracies in integration due distortions/sources of inaccuracy present in ED data collection are expected here.

PLAT341_ALERT_3_B Low Bond Precision on C-C Bonds .... 0.01975 Ang.

Author response: The missing wedge of data in electron diffraction measurements often limits data completeness in certain regions of reciprocal space. This is likely to reduce precision of some bond lengths.

Zn(II)-histidine (dry cryogenic temperature MicroED)

A-level alerts

PLAT029 ALERT 3 A diffrn measured fraction theta full value Low . 0.836 Why?

Author response: The missing wedge of data in electron diffraction measurements often limits data completeness in certain regions of reciprocal space.

B-level alerts

RINTA01_ALERT_3_B The value of Rint is greater than 0.18
Rint given 0.181

PLAT020_ALERT_3_B The Value of Rint is Greater Than 0.12 .... 0.181 Report

Author response: The data comes from electron diffraction measurements. Some inaccuracies in integration due both to distortions/sources of inaccuracy present in ED data collection are expected here, leading to inflation of Rint values.

```
PLAT911_ALERT_3_B Missing FCF Refl Between Thmin & STh/L=    0.600      127 Report
          3  8  0,   3  8  1,   6  6  4,   0  0  8,   0  1  8,   0  1  9,
          1  1  9,   2  8  9,   0  1 10,   1  1 10,   0  1 11,   1  1 11,
          0  0 12,   0  1 12,   1  1 12,   0  1 13,   1  1 13,   0  1 14,
          1  1 14,   0  1 15,   1  1 15,   0  0 16,   0  1 16,   1  1 16,
          0  2 16,   0  1 17,   1  1 17,   0  2 17,   1  2 17,   0  1 18,
            (  97 More Missing: see the .ckf listing file)
```

Author response: The missing wedge of data in electron diffraction measurements often limits data completeness in certain regions of reciprocal space.

3PhCOOK

A-level alerts

THETM01_ALERT_3_A The value of sine(theta_max)/wavelength is less than 0.550
Calculated sin(theta_max)/wavelength = 0.4999

Author Response: The data comes from electron diffraction measurement collected at room temperature for crystals suspended in mother liquor. As such, diffraction measurements past 1.0 Angstrom resolution (sin(theta_max/wavelength = 0.5)) on these crystals were not reliably of sufficient quality for inclusion in the dataset, though the 1.0 Angstrom dataset was of sufficient quality to phase by direct methods and produce an accurate structure solution.

PLAT029_ALERT_3_A _diffrn_measured_fraction_theta_full value Low . 0.635 Why?

Author Response: Needle-shaped microcrystals showed preferential orientation on the TEM grid, giving access to a fraction of the reciprocal space only, even after merging multiple datasets.

PLAT088_ALERT_3_A Poor Data / Parameter Ratio .... 5.34 Note

Author Response: Needle-shaped microcrystals showed preferential orientation on the TEM grid, giving access to a fraction of the reciprocal space only, even after merging multiple datasets.

B-level alerts

RINTA01_ALERT_3_B The value of Rint is greater than 0.18
Rint given 0.198
PLAT020_ALERT_3_B The Value of Rint is Greater Than 0.12 .... 0.198 Report
PLAT084_ALERT_3_B High wR2 Value (i.e. > 0.25) .... 0.40 Report

Author response: The data comes from electron diffraction measurement collected at room temperature for crystals suspended in mother liquor. Some inaccuracies in integration due both to distortions/sources of inaccuracy present in ED data collection, and due to measurement of crystals in a liquid medium, are expected here.

PLAT331_ALERT_2_B Small Aver Phenyl C-C Dist C75 --C80 . 1.35 Ang.
PLAT340_ALERT_3_B Low Bond Precision on C-C Bonds .... 0.02426 Ang.
PLAT355_ALERT_3_B Long O-H (X0.82,N0.98A) O4 - H4 . 1.10 Ang.

Author response: The missing wedge of data in electron diffraction measurements often limits data completeness in certain regions of reciprocal space. This is likely to reduce precision of some bond lengths.

```
PLAT911_ALERT_3_B Missing FCF Refl Between Thmin & STh/L=   0.500      2419 Report
            1 1 0,  0 2 0,  1 2 0, -1 3 0,  0 3 0,  1 3 0,
            2 3 0,  3 3 0, -1 4 0,  0 4 0,  1 4 0,  2 4 0,
            3 4 0,  4 4 0,  8 4 0, -1 5 0,  0 5 0,  1 5 0,
            2 5 0,  3 5 0,  4 5 0, -2 6 0, -1 6 0,  0 6 0,
            1 6 0,  2 6 0,  3 6 0,  4 6 0,  5 6 0, -2 7 0,
              ( 2389 More Missing: see the .ckf listing file)
```

Author response: The missing wedge of data in electron diffraction measurements often limits data completeness in certain regions of reciprocal space.

Ceftazidime pentahydrate (LC-MicroED)

A-level alerts

RINTA01_ALERT_3_A The value of Rint is greater than 0.25
Rint given 0.269

PLAT020_ALERT_3_A The Value of Rint is Greater Than 0.12 .... 0.269 Report

Author response: The data comes from electron diffraction measurement collected at room temperature for crystals suspended in mother liquor. Some inaccuracies in integration due both to distortions/sources of inaccuracy present in ED data collection, and due to measurement of crystals in a liquid medium, are expected here.

PLAT029_ALERT_3_A _diffrn_measured_fraction_theta_full value Low . 0.821 Why?

Author response: The missing wedge of data in electron diffraction measurements often limits data completeness in certain regions of reciprocal space, as was the case for these orientation-biased plate-shaped crystals on the TEM grid.

B-level alerts

PLAT084_ALERT_3_B High wR2 Value (i.e. > 0.25) .... 0.41 Report

Author response: The data comes from electron diffraction measurement collected at room temperature for crystals suspended in mother liquor. Some inaccuracies in integration due both to distortions/sources of inaccuracy present in ED data collection, and due to measurement of crystals in a liquid medium, are expected here.

PLAT306_ALERT_2_B Isolated Oxygen Atom (H-atoms Missing ?) .... O8 Check

And 4 other PLAT306 Alerts

Less ...

PLAT306_ALERT_2_B Isolated Oxygen Atom (H-atoms Missing ?) .... O9 Check
PLAT306_ALERT_2_B Isolated Oxygen Atom (H-atoms Missing ?) .... O10 Check
PLAT306_ALERT_2_B Isolated Oxygen Atom (H-atoms Missing ?) .... O11 Check
PLAT306_ALERT_2_B Isolated Oxygen Atom (H-atoms Missing ?) .... O12 Check

Author response: Clear evidence of H-atoms in the electrostatic potential map attached to coordinated water molecules was not detected, so hydrogen atoms were not modeled on water molecules.

PLAT340_ALERT_3_B Low Bond Precision on C-C Bonds .... 0.02731 Ang.
PLAT430_ALERT_2_B Short Inter D...A Contact O1 ..O10 . 2.58 Ang.
1/2+x,1/2-y,2-z = 2_557 Check
PLAT430_ALERT_2_B Short Inter D...A Contact O2 ..O9 . 2.72 Ang.
-1/2+x,1/2-y,2-z = 2_457 Check

And 2 other PLAT430 Alerts

Less ...

PLAT430_ALERT_2_B Short Inter D...A Contact O3 ..O12 . 2.73 Ang.
x,y,z = 1_555 Check
PLAT430_ALERT_2_B Short Inter D...A Contact O4 ..O12 . 2.84 Ang.
-1/2+x,1/2-y,2-z = 2_457 Check
PLAT430_ALERT_2_B Short Inter D...A Contact O6 ..O12 . 2.59 Ang.
1/2+x,1/2-y,2-z = 2_557 Check

And 2 other PLAT430 Alerts

Less ...

PLAT430_ALERT_2_B Short Inter D...A Contact O8 ..O9 . 2.71 Ang.
x,y,z = 1_555 Check
PLAT430_ALERT_2_B Short Inter D...A Contact O10 ..O11 . 2.80 Ang.
2-x,-1/2+y,3/2-z = 3_746 Check
PLAT430_ALERT_2_B Short Inter D...A Contact O11 ..N5 . 2.76 Ang.
x,y,z = 1_555 Check

Author response: The missing wedge of data in electron diffraction measurements often limits data completeness in certain regions of reciprocal space. This is likely to reduce precision of some bond lengths.

PLAT911_ALERT_3_B Missing FCF Refl Between Thmin & STh/L= 0.600 478 Report
3 0 1, 0 0 4, 1 0 5, 0 1 5, 0 0 6, 1 0 6,
2 0 6, 0 1 6, 1 0 7, 0 1 7, 0 0 8, 1 0 8,
0 1 8, 1 1 8, 1 0 9, 3 0 9, 0 1 9, 1 1 9,
0 0 10, 1 0 10, 2 0 10, 0 1 10, 1 1 10, 0 2 10,
1 0 11, 2 0 11, 0 1 11, 1 1 11, 0 2 11, 1 2 11,
(448 More Missing: see the .ckf listing file)

Author response: The missing wedge of data in electron diffraction measurements often limits data completeness in certain regions of reciprocal space, as was the case for these orientation-biased plate-shaped crystals on the TEM grid.

Ceftazidime (monohydrate, dry room-temperature MicroED)

A-level alerts

```
PLAT082_ALERT_2_A High R1 Value ................................     0.25 Report
PLAT084_ALERT_3_A High wR2 Value (i.e. > 0.25) ..................     0.56 Report
```

Author response: The data comes from electron diffraction measurement collected at room temperature. Some inaccuracies in integration due distortions/sources of inaccuracy present in ED data collection are expected here.

B-level alerts

```
PLAT340_ALERT_3_B Low Bond Precision on  C-C Bonds ..............      0.04 Ang.
PLAT369_ALERT_2_B Long   C(sp2)-C(sp2) Bond  C4      - C5      .      1.64 Ang.
PLAT369_ALERT_2_B Long   C(sp2)-C(sp2) Bond  C9      - C10     .      1.57 Ang.
PLAT430_ALERT_2_B Short Inter D...A Contact  O1      ..O7      .      2.65 Ang.
                            1/2-x,3-y,1/2+z  =     2_585 Check
PLAT430_ALERT_2_B Short Inter D...A Contact  O1      ..N5      .      2.87 Ang.
                            1/2-x,2-y,1/2+z  =     2_575 Check
PLAT430_ALERT_2_B Short Inter D...A Contact  O2      ..N5      .      2.77 Ang.
                           -1/2+x,5/2-y,2-z  =     4_477 Check
PLAT430_ALERT_2_B Short Inter D...A Contact  O6      ..N1      .      2.78 Ang.
                            1/2-x,3-y,-1/2+z  =    2_584 Check
```

Author response: The missing wedge of data in electron diffraction measurements often limits data completeness in certain regions of reciprocal space. This is likely to reduce precision of some bond lengths.

```
PLAT911_ALERT_3_B Missing FCF Refl Between Thmin & STh/L=  0.600      370 Report
        2  0  0,   0  0  4,   1  0  4,   0  1  4,   1  0  5,   4  0  5,
        0  1  5,   0  0  6,   1  0  6,   0  1  6,   2  1  6,   1  0  7,
        0  1  7,   2  1  7,   0  2  7,   1  2  7,   0  3  7,   1  3  7,
        0  5  7,   0  0  8,   1  0  8,   2  0  8,   0  1  8,   1  1  8,
        0  2  8,   1  2  8,   0  4  8,   1  0  9,   2  0  9,   0  1  9,
          (  340 More Missing: see the .ckf listing file)
```

Author response: The missing wedge of data in electron diffraction measurements often limits data completeness in certain regions of reciprocal space.

d-AVAAGA (LC-MicroED)

A-level alerts

ATOM007_ALERT_1_A _atom_site_aniso_label is missing
Unique label identifying the atom site.
PLAT201_ALERT_2_A Isotropic non-H Atoms in Main Residue(s) .... 32 Report
O1 O2 O3 O4 O5 O6 O7 N1
N2 N3 N4 N5 N6 C1 C2 C3
C4 C5 C6 C7 C8 C9 C10 C11
C12 C13 C14 C15 C16 C17 C18 C19

Author Response: Only isotropic refinement of ADPs was performed, as anisotropic refinement resulted in physically unrealistic stretched or flattened atomic ellipsoids.

PLAT082_ALERT_2_A High R1 Value .... 0.26 Report
PLAT084_ALERT_3_A High wR2 Value (i.e. > 0.25) .... 0.56 Report

Author Response: The data comes from electron diffraction measurement collected at room temperature for crystals suspended in mother liquor. Some inaccuracies in integration due both to distortions/sources of inaccuracy present in ED data collection, and due to measurement of crystals in a liquid medium, are expected here.

PLAT029_ALERT_3_A _diffrn_measured_fraction_theta_full value Low . 0.925 Why?

Author Response: Needle-shaped microcrystals showed preferential orientation on the TEM grid, giving access to a fraction of the reciprocal space only, even after merging multiple datasets.

B-level alerts

RINTA01_ALERT_3_B The value of Rint is greater than 0.18
Rint given 0.234
PLAT020_ALERT_3_B The Value of Rint is Greater Than 0.12 .... 0.234 Report

Author Response: The data comes from electron diffraction measurement collected at room temperature for crystals suspended in mother liquor. Some inaccuracies in integration due both to distortions/sources of inaccuracy present in ED data collection, and due to measurement of crystals in a liquid medium, are expected here. Merging of 10 partial datasets was necessary to achieve sufficient completeness under these conditions, contributing to an inflation of Rint.

PLAT242_ALERT_2_B Low 'MainMol' Ueq as Compared to Neighbors of C5 Check

Author response: Some resolution anisotropy resulting from limited data completeness in certain regions of reciprocal space, exacerbated by the preferential orientation of these needle-shaped crystals on the TEM grid, likely introduced some errors in the consistency of certain atomic B-factors relative to those of neighboring atoms. The elemental assignment of C5 has been checked and deemed accurate.

PLAT340_ALERT_3_B Low Bond Precision on C-C Bonds .... 0.03077 Ang.
PLAT360_ALERT_2_B Short C(sp3)-C(sp3) Bond C12 - C13 . 1.31 Ang.
PLAT413_ALERT_2_B Short Inter XH3 .. XHn H02C ..H03D . 1.98 Ang.
1-x,-1/2+y,1/2-z = 3_645 Check

Author response: The missing wedge of data in electron diffraction measurements often limits data completeness in certain regions of reciprocal space. This is likely to reduce precision of some bond lengths.

PLAT911_ALERT_3_B Missing FCF Refl Between Thmin & STh/L= 0.600 175 Report

```
0 0 4,  1 0 4,  0 1 5,  0 1 6,  0 1 7,  0 2 7,
0 1 9,  0 2 9,  0 2 10,  0 2 11,  0 2 12,  1 0 13,
0 2 13,  1 2 13,  0 3 13,  0 2 14,  1 2 14,  0 3 14,
1 2 15,  0 3 15,  1 3 15,  0 4 15,  0 3 16,  1 3 16,
0 3 17,  1 3 17,  0 4 17,  0 3 18,  1 3 18,  0 4 18,
  ( 145 More Missing: see the .ckf listing file)
```

Author response: The missing wedge of data in electron diffraction measurements often limits data completeness in certain regions of reciprocal space.

d-AVAAGA (dry cryogenic temperature MicroED)

A-level alerts

PLAT029_ALERT_3_A _diffrn_measured_fraction_theta_full value Low . 0.910 Why?

Author Response: Needle-shaped microcrystals showed preferential orientation on the TEM grid, giving access to a fraction of the reciprocal space only, even after merging multiple datasets.

PLAT084_ALERT_3_A High wR2 Value (i.e. > 0.25) .... 0.46 Report

Author response: The data comes from electron diffraction measurements. Some inaccuracies in integration due distortions/sources of inaccuracy present in conventional ED data collection are expected here.

B-level alerts

RINTA01_ALERT_3_B The value of Rint is greater than 0.18
Rint given 0.214
PLAT020_ALERT_3_B The Value of Rint is Greater Than 0.12 .... 0.214 Report

Author response: The data comes from electron diffraction measurements. Some inaccuracies in integration due both to distortions/sources of inaccuracy present in ED data collection are expected here, leading to inflation of Rint values

PLAT082_ALERT_2_B High R1 Value .... 0.19 Report

Author response: The data comes from electron diffraction measurements. Some inaccuracies in integration due distortions/sources of inaccuracy present in conventional ED data collection are expected here.

PLAT340_ALERT_3_B Low Bond Precision on C-C Bonds .... 0.02385 Ang.
PLAT362_ALERT_2_B Short C(sp3)-C(sp2) Bond C17 - C19 . 1.30 Ang.

Author response: The missing wedge of data in electron diffraction measurements often limits data completeness in certain regions of reciprocal space. This is likely to reduce precision of some bond lengths.

```
PLAT911_ALERT_3_B Missing FCF Refl Between Thmin & STh/L=   0.600      196 Report
           0 13  3,  0  0  4,  0  0  6,  0  1  7,  0  0  8,  0  1  9,
           0  0 10,  0  1 10,  0  1 11,  0  0 12,  1  0 12,  0  1 12,
           0  1 13,  0  0 14,  0  1 14,  0  2 14,  0  1 15,  0  0 16,
           1  0 16,  0  1 16,  1  1 16,  1  0 17,  0  1 17,  0  0 18,
           1  0 18,  0  1 18,  0  2 18,  1  0 19,  2  0 19,  0  1 19,
             (  166 More Missing: see the .ckf listing file)
```

Author response: The missing wedge of data in electron diffraction measurements often limits data completeness in certain regions of reciprocal space.

Supplementary references

(1) Kabsch, W. *XDS*. *Acta Crystallogr D Biol Crystallogr* **2010**, *66* (2), 125–132. https://doi.org/10.1107/S0907444909047337.

(2) Kabsch, W. Integration, Scaling, Space-Group Assignment and Post-Refinement. *Acta Crystallogr D Biol Crystallogr* **2010**, *66* (2), 133–144. https://doi.org/10.1107/S0907444909047374.

(3) Sheldrick, G. M. A Short History of *SHELX*. *Acta Crystallogr A Found Crystallogr* **2008**, *64* (1), 112–122. https://doi.org/10.1107/S0108767307043930.

(4) Sheldrick, G. M. Crystal Structure Refinement with *SHELXL*. *Acta Crystallogr C Struct Chem* **2015**, *71* (1), 3–8. https://doi.org/10.1107/S2053229614024218.

(5) Hübschle, C. B.; Sheldrick, G. M.; Dittrich, B. *ShelXle* : A Qt Graphical User Interface for *SHELXL*. *J Appl Crystallogr* **2011**, *44* (6), 1281–1284. https://doi.org/10.1107/S0021889811043202.

(6) McCoy, A. J.; Grosse-Kunstleve, R. W.; Adams, P. D.; Winn, M. D.; Storoni, L. C.; Read, R. J. *Phaser* Crystallographic Software. *J Appl Crystallogr* **2007**, *40* (4), 658–674. https://doi.org/10.1107/S0021889807021206.

(7) Adams, P. D.; Afonine, P. V.; Bunkóczi, G.; Chen, V. B.; Davis, I. W.; Echols, N.; Headd, J. J.; Hung, L.-W.; Kapral, G. J.; Grosse-Kunstleve, R. W.; McCoy, A. J.; Moriarty, N. W.; Oeffner, R.; Read, R. J.; Richardson, D. C.; Richardson, J. S.; Terwilliger, T. C.; Zwart, P. H. *PHENIX* : A Comprehensive Python-Based System for Macromolecular Structure Solution. *Acta Crystallogr D Biol Crystallogr* **2010**, *66* (2), 213–221. https://doi.org/10.1107/S0907444909052925.
